# Beyond cleaved small RNA targets: unraveling the complexity of plant RNA degradome data

**DOI:** 10.1186/1471-2164-15-15

**Published:** 2014-01-10

**Authors:** Cheng-Yu Hou, Ming-Tsung Wu, Shin-Hua Lu, Yue-Ie Hsing, Ho-Ming Chen

**Affiliations:** 1 Agricultural Biotechnology Research Center, Academia Sinica, Taipei 11529, Taiwan; 2 Institute of Plant and Microbial Biology, Academia Sinica, Taipei 11529, Taiwan; 3 Institute of Plant Biology, National Taiwan University, Taipei 10617, Taiwan

**Keywords:** Degradome, RNA degradation, RNA motif, RNA-binding protein, Sequencing artifact

## Abstract

**Background:**

Degradation is essential for RNA maturation, turnover, and quality control. RNA degradome sequencing that integrates a modified 5′-rapid amplification of cDNA ends protocol with next-generation sequencing technologies is a high-throughput approach for profiling the 5′-end of uncapped RNA fragments on a genome-wide scale. The primary application of degradome sequencing has been to identify the truncated transcripts that result from endonucleolytic cleavage guided by microRNAs or small interfering RNAs. As many pathways are involved in RNA degradation, degradome data should contain other RNA species besides the cleavage remnants of small RNA targets. Nevertheless, no systematic approaches have been established to explore the hidden complexity of plant degradome.

**Results:**

Through analyzing Arabidopsis and rice RNA degradome data, we recovered 11 short motifs adjacent to predominant and abundant uncapped 5′-ends. Uncapped ends associated with several of these short motifs were more prevalent than those targeted by most miRNA families especially in the 3′ untranslated region of transcripts. Through genome-wide analysis, five motifs showed preferential accumulation of uncapped 5′-ends at the same position in Arabidopsis and rice. Moreover, the association of uncapped 5′-ends with a CA-repeat motif and a motif recognized by Pumilio/Fem-3 mRNA binding factor (PUF) proteins was also found in non-plant species, suggesting that common mechanisms are present across species. Based on these motifs, potential sources of RNA ends that constitute degradome data were proposed and further examined. The 5′-end of small nucleolar RNAs could be precisely captured by degradome sequencing. Position-specific enrichment of uncapped 5′-ends was seen upstream of motifs recognized by several RNA binding proteins especially for the binding site of PUF proteins. False uncapped 5′-ends produced from capped transcripts through non-specific PCR amplification were common artifacts among degradome datasets.

**Conclusions:**

The complexity of plant RNA degradome data revealed in this study may contribute to the alternative applications of degradome in RNA research.

## Background

Degradation plays vital roles in RNA maturation, turnover, and quality control. Almost all RNA species are transcribed longer before becoming functional forms and require the removal of extra sequences in the termini (5′ or 3′ processing) or internal regions (splicing). Mature 5′ RNA ends generally possess a triphosphate or a 7-methylguanosine cap, whereas mature 3′ RNA ends possess a poly(A) tail or a stem-loop structure. Loss of these specific features stimulates RNA turnover [1]. Defective RNAs containing a premature stop codon, lacking an in-frame stop codon or carrying stalled ribosomes are eliminated by mRNA-surveillance pathways [2-5]. RNA degradation can proceed from the 5′-end, the 3′-end, or internally with 5′-to-3′ exoribonucleases, 3′-to-5′ exoribonucleases, and endoribonuclease, respectively. Maturation of ribosomal RNAs (rRNAs), transfer RNAs, small nuclear RNAs (snRNAs) and small nucleolar RNAs (snoRNAs) relies on the delicate cooperation of exoribonucleases and endoribonuclease. *Cis*-elements on mRNAs can trigger endonucleolytic cleavage or deadenylation and therefore destabilize RNA. The exosome is the major component in versatile RNA maturation and surveillance pathways [6]. Some exoribonucleases have dual functions, and can degrade entire transcripts for some RNA species and define the termini of mature RNAs for other RNA species. For instance, the yeast 5′-to-3′ exoribonuclease Rat1 participates in the degradation of unspliced pre-mRNAs as well as the formation of snoRNA 5′-ends [7,8].

Small regulatory RNAs (20–24 nt) such as microRNAs (miRNAs) and small interfering RNAs (siRNAs) can initiate endonucleolytic cleavage in the middle of highly complementary target sites on long transcripts [9]. Small RNA-guided cleavage is mediated by Argonaute proteins which possess small RNA binding domains and endonuclease domains [10]. The 3′ cleavage remnant of some plant miRNA targets is the substrate of a 5′-to-3′ exoribonuclease, XRN4/EIN5 [11]. Specific cleavage sites initiated by small RNAs are frequently validated using a modified 5′-rapid amplification of cDNA ends (5′ RACE) protocol that skips enzyme treatment for the removal of the 5′ phosphate and the capping structure [12]. With this modification, 5′ RNA adaptors can only ligate to RNA molecules without a cap structure but with a monophosphate at the 5′-end which are the typical products of small RNA-guided cleavage, thus preventing sequencing of full-length mRNAs with a cap structure. Advances in high-throughput sequencing technologies have enabled genome-wide surveys of uncapped RNA molecules and parallel validation of numerous small RNA targets. High-throughput methods for profiling uncapped RNA termini have been established independently by several groups and are known variously as degradome sequencing, parallel analysis of RNA ends (PARE) and genome-wide mapping of uncapped transcripts (GMUCT) [13-15]. The three approaches all start with the enrichment of poly(A) RNA for the ligation of 5′ RNA adaptors but use either enzyme digestion (PARE and degradome sequencing) or sonication (GMUCT) to produce small fragments suitable for sequencing. This methodology has been widely applied to budding yeast, Arabidopsis, rice, maize, grape, soybean and poplar as well as mammals including mice and humans for the identification of miRNA targets or mRNA decay intermediates [13-25].

Current degradome data analysis mainly focuses on the identification of small RNA targets. Several tools such as CleaveLand, SeqTar, and PAREsnip have been developed to fulfill this purpose by pairing sequences flanking uncapped 5′-ends with small RNA sequences [26-28]. The tools have been successfully used to uncover known and new miRNA targets in many organisms. As RNA is constitutively synthesized and subject to bulk or specific degradation, the degradome should represent a complex collection of intermediates produced during RNA maturation or decay. A previous analysis of mouse degradome data revealed miRNA-guide cleavage as well as miRNA-independent events including a group of transcripts sharing a CA-repeat motif within the truncated site [20]. Although degradome data could facilitate the study of RNA degradation beyond the RNA silencing pathways, systematic approaches that dissect degradome data to elucidate mechanisms independent of small RNA regulation have not been established.

In this study, we developed a new pipeline for the analysis of RNA degradome data without a prior assumption of small RNA-guided cleavage to investigate potential mechanisms underlying the formation of uncapped 5′-ends. Our analysis revealed short sequence motifs adjacent to uncapped 5′-ends that were conserved across different degradome libraries and species. Based on sequence similarity and the unique location of these motifs, we have proposed potential routes that may contribute to the complexity and the quality of plant RNA degradome data.

## Results and discussion

### Analysis of motifs associated with predominant uncapped 5′-ends

Presumably the uncapped 5′-ends in degradome datasets are a mixture of the randomly and specifically degraded products of various degradation pathways. In this study, we focused on predominant uncapped 5′-ends which had significantly higher abundance than those produced at nearby positions. We hypothesized that short RNA motifs which are not miRNA target sites might be associated with the formation of dominantly truncated 5′-ends in plant degradome data as reported in mouse data [20]. To explore this possibility in plants, we analyzed two Arabidopsis PARE libraries, TWF (Col-0 inflorescence) and Tx4F (*xrn4* inflorescence), and four rice PARE libraries, INF9311a (wild-type 93–11 inflorescence), INF939 (wild-type Nipponbare inflorescence), SC938 (wild-type Nipponbare seedlings) and NPBs (wild-type Nipponbare seedlings) [14,21,23,25]. For Arabidopsis, in addition to PARE libraries, three libraries generated by degradome sequencing, AxIDT (Col-0 inflorescence, oligo dT primed), AxIRP (Col-0 inflorescence, random primed), and AxSRP(Col-0 seedling, random primed), and two libraries generated by the GMUCT method, Col-0 (Col-0 inflorescence) and *ein5* (*ein5* inflorescence), were also included in the analysis [13,15]. We first removed reads of low complexity which had multiple hits in the genome and interfered with motif analysis. Since different degradation mechanisms may prefer acting in distinct genomic regions, we thus classified uncapped reads according to their genomic origin, the 5′ or 3′ untranslated region (UTR), coding sequence (CDS), intergenic region (IGR), or intron, by the use of Bowtie with zero mismatch [29]. Uncapped 5′-ends defined by deep sequencing were selected for motif analysis based on two criteria. First, an uncapped 5′-end was selected if the read number from that specific position plus the positions 1-nt upstream and 1-nt downstream of it constituted 50% of the total reads occurring in a 21-nt window symmetrically flanking the 5′-end. All uncapped 5′-ends that passed this criterion were then subjected to statistical evaluation using a binomial test with the following Equation

$$P\left(x\right)=\left({}_{x}^{n}\right)q^{x}{\left(1-q\right)}^{n-x}$$

where *x* was the read number of an uncapped 5′-end while *n* was the total read number occurring within the 21-nt window symmetrically flanking it. Assuming that each position within the 21-nt window has the same probability to produce uncapped 5′-ends, the probability of a read occurring at one position, *q* in the equation, was assigned as 1/21. Uncapped 5′-ends with a *P*-value less than 10^-5^ were selected for motif analysis with the MEME suite. The MEME suite is a commonly used program that identifies motifs within a group of DNA or protein sequences that share similar properties [30]. More than 1000 uncapped 5′-ends passed the statistical test in some genomic regions for some libraries (Additional file 1: Table S1). In this case, the uncapped 5′-ends were ranked according to abundance and the top 1000 most abundant ends were selected. To focus on mechanisms independent of miRNA regulation, uncapped 5′-ends corresponding to the cleavage sites initiated by known Arabidopsis and rice miRNAs were filtered before motif analysis. The numbers of unique reads of each library and uncapped 5′-ends that passed the statistical test are shown in Additional file 1: Table S1. Among the uncapped 5′-ends passing the statistical test, the number of unique ends resulting from miRNA-guided cleavage and the number of unique ends used in motif analysis are also summarized in Additional file 1: Table S1.

**Figure 1:**
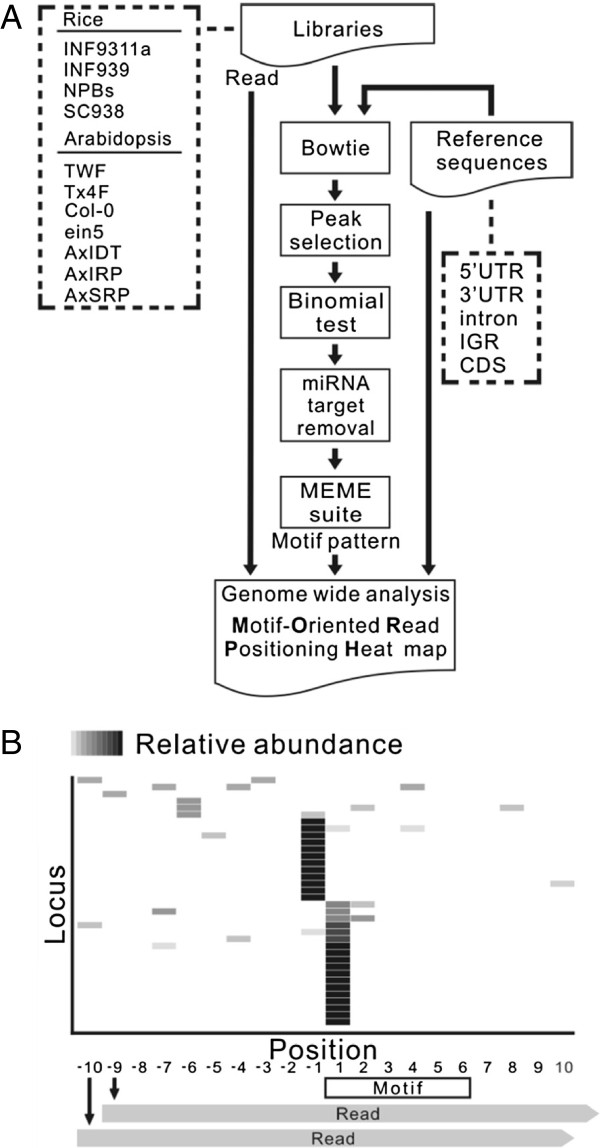
**Analysis schemas for motifs associated with uncapped 5′-ends.** The analysis pipeline for identifying motifs associated with uncapped 5′-ends **(A)**. Uncapped 5′-ends were first classified by the genomic region where they were produced by mapping with Bowtie. Selected uncapped 5′-ends representing as major peaks were further filtered with the binomial test. After filtering known miRNA targets, all the uncapped 5′-ends which passed the threshold of the binomial test or the top 1000 most abundant ends in each genomic region were subjected to motif analysis with the MEME suite. The spatial relationship between motifs and uncapped reads on a genome-wide scale was further explored by MORPH **(B)**. All loci containing a candidate motif in a specific genomic region were first identified and then clustered based on the distribution of normalized reads in a 20-nt region flanking the motif. With the first nucleotide of the motif set as 1, positions upstream of the motif are indicated as negative values and positions downstream are indicated as positive values. The position of an uncapped read was determined by its 5′-end and the number of uncapped reads at each position was normalized to the total read number within the 20-nt region and represented as a heat map.

Motifs present in a 50-nt region spanning 25-nt upstream and 25-nt downstream of selected uncapped 5′-end were further filtered according to the statistical significance of the motif, the *E*-value generated by the MEME suite, and the distribution of motif sites relative to the uncapped 5′-end. This study only focused on the motifs with *E*-values smaller than 1 and those were predominantly found at a specific position where the occurrences of the motif plus the occurrences at the positions 1-nt upstream and 1-nt downstream of it constituted at least 50% of all motif sites found within the 50-nt region. To examine whether motifs identified by the MEME suite could be extended or belong to part of unknown small RNA target sites which usually span 21 nt, we then aligned the sequences flanking the selected motifs. Motifs identified in different libraries and genomic regions were manually merged into groups based on sequence homology. A representative motif for each group was then generated manually. To gain more insight into these motifs, we then conducted reverse analysis of the occurrences of uncapped reads surrounding every candidate motif on a genome-wide scale using a cluster heat map that we named motif-oriented read positioning heat map (MORPH). Schemas illustrating the analysis pipeline and the concept of MORPH are shown in Figure 1A and B.

### Position-specific motifs surrounding predominant uncapped 5′-ends

The number of uncapped 5′-ends passing the statistical test was highly variable among the different degradome libraries (Additional file 1: Table S1). This might be explained by the total read number of each library or the degree of RNA integrity for each sequencing sample. The uncapped 5′-ends initiated by known miRNAs represented less than 2% of the total unique ends passing the statistical test which suggests that miRNA-independent mechanisms may contribute significantly to the formation of predominant uncapped 5′-ends (Additional file 1: Table S1).

In addition to a motif group corresponding to rice miR2118 target sites which are associated with the production of secondary siRNAs from hundreds of rice loci in the IGR [31], 11 motif groups were recovered from the analyses of 11 Arabidopsis and rice degradome libraries (Table 1). Motifs 1, 2 and 9 were identified in both species, suggesting that common mechanisms independent of miRNA-guided cleavage for the formation of predominant uncapped 5′-ends are present across species. Notably, motifs within a group which were derived from independent analyses of different genomic regions, libraries, or species were dominantly located at neighboring positions relative to the uncapped 5′-end. For example, motifs within group 2 were mainly at the downstream 3^rd^ and 4^th^ positions relative to the uncapped 5′-end (Table 1). On the other hand, motifs 9, 10, and 11 were all present immediately upstream of the uncapped 5′-end and were demonstrated to be potential artifacts produced during library construction (see the section below for details). Surprisingly, motif 4, a CA-repeat sequence, was identical to the motif reported previously from the analysis of mouse degradome data and was present at the same position relative to the uncapped 5′-end (Additional file 2: Figure S1) [20]. The fixed distance of motifs to the uncapped 5′-end across species and libraries strengthens the hypothesis that these motifs are associated with the formation of uncapped 5′-ends.

**Table 1:** Motifs identified from the analysis of predominant uncapped 5′-ends in Arabidopsis and rice degradome libraries

**Group**	**Library**^**a**^	**Region**^**b**^	**Motif**^**c**^	***E*-value**^**d**^	**Position**^**e**^	**Site**^**f**^
1 RTGATGA	TWF (At)	IGR	KRTGATGA	7.60E-22	5	28(1000)
	Tx4F (At)	intron	RATGATGA	2.00E-06	4	13(770)
	INF9311a (Os)	intron	RTGATGA	7.70E-05	6	20(817)
	NPBs (Os)	IGR	DRTGATGA	6.40E-24	5	37(1000)
	NPBs (Os)	intron	RTGATGAD	2.00E-11	6	20(1000)
2 TGTAHAKA	TWF (At)	3′UTR	TGTAHATA	2.00E-82	4	110(1000)
	Tx4F (At)	3′UTR	TGTAHAKW	4.40E-52	3	72(1000)
	INF9311a (Os)	intron	YTGTAMAK	1.10E-21	3	55(817)
	INF9311a (Os)	CDS	TGTACAG	1.20E-07	4	27(1000)
	INF9311a (Os)	3′UTR	YTGTAHAK	1.00E-376	3	320(1000)
	INF939 (Os)	3′UTR	HTGTAMWK	3.50E-135	3	119(1000)
	NPBs (Os)	3′UTR	YTGTAMAK	1.30E-164	3	174(1000)
	NPBs (Os)	IGR	TGTAHAKW	5.70E-26	4	62(1000)
	NPBs (Os)	intron	TGTACAKA	1.30E-22	4	55(1000)
3 AATAAA	Tx4F (At)	3′UTR	AAYAAARV	2.30E-10	4	60(1000)
4 CACACACA	INF939 (Os)	CDS	CACACACA	1.10E-01	-1	15(599)
	INF939 (Os)	3′UTR	CACACACA	2.70E-01	-1	9(1000)
5 ATGTATGT	Col-0 (At)	3′UTR	ATGTATGT	1.70E-38	-1	103(499)
6 GTCTRGTG	Tx4F (At)	IGR	GTCTRGTG	6.10E-05	16	12(1000)
7 CAGAC	NPBs (Os)	3′UTR	MCAGAC	5.60E-02	1	40(1000)
8 AAAAAAAA	INF9311a (Os)	IGR	AAAAAAAA	2.40E-07	12	16(1000)
9 GTCCGAC	Tx4F (At)	CDS	AGTCCGAC	9.20E-21	-8	35(1000)
	INF9311a (Os)	CDS	AGYCCGAC	1.50E-64	-8	81(1000)
	INF939 (Os)	CDS	AGTCCGAC	4.60E-31	-8	60(599)
	INF939 (Os)	3′UTR	RSYCCRAC	1.30E-07	-8	59(1000)
	NPBs (Os)	CDS	ASKCCGAC	8.90E-258	-8	298(1000)
	NPBs (Os)	3′UTR	VBCCGACH	8.90E-51	-7	85(1000)
	NPBs (Os)	intron	SKCCGACH	1.10E-09	-7	30(1000)
10 GATCCAAC	AxIDT (At)	3′UTR	GATCCAAM	4.50E-03	-8	10(793)
	AxIRP (At)	CDS	GRTCCAAC	1.00E-126	-8	121(1000)
	AxIRP (At)	5′UTR	RATCCAAC	5.00E-19	-8	49(1000)
	AxIRP (At)	intron	GRTCCAAC	7.10E-01	-8	18(1000)
	AxSRP (At)	CDS	GATCCAAC	8.40E-40	-8	45(1000)
	AxSRP (At)	5′UTR	GATCCAAC	9.80E-07	-8	22(1000)
	AxSRP (At)	intron	GATCCAAC	3.70E-01	-8	15(1000)
11 GACGATC	Col-0 (At)	3′UTR	VMGACGAT	3.40E-02	-9	15(499)
	*ein5* (At)	3′UTR	CGACGATY	3.20E-06	-8	23(153)
	*ein5* (At)	CDS	SGACGWTY	1.50E-03	-8	17(476)

^a^At: Arabidopsis; Os: rice.

^b^IGR, UTR and CDS indicate the intergenic region, the untranslated region and the coding sequence, respectively.

^c^Syntax for multiple bases: B = C/G/T, D = A/G/T, H = A/C/T, K = G/T, M = A/C, R = A/G, S = G/C, V = A/C/G, W = A/T, Y = C/T.

^d^*E*-value is the estimated number of (equally or more significant) motifs that one would expect to find by chance if the input sequences were shuffled.

^e^Position indicates the predominant position of the first nucleotide of the motif relative to the uncapped 5′-end revealed by deep sequencing which was set to 1. Upstream positions are indicated as negative values and downstream positions are indicated as positive values.

^f^The numbers indicate sites possessing the indicated motif at the specific position among the number of input sequences (in parentheses) for MEME analysis.

The majority of motifs could be recovered from the 3′ UTR which is in contrast to that most plant miRNAs target the CDS (Table 1). For most miRNAs of Arabidopsis and rice, targets of a single miRNA family do not exceed 20 [28]. However, motifs identified in this study were often associated with more than 20 sites among 1000 or fewer uncapped 5′-ends used in MEME analysis. Motif 2 was the most significant example, being found in more than 100 sites among 1000 uncapped 5′-ends in the 3′ UTR for three rice libraries (Table 1). The results of motif analyses thus suggest that mechanisms underlying the formation of uncapped 5′-ends containing these short motifs might play prominent roles in the production of predominant uncapped 5′-ends in addition to miRNA regulation especially in the 3′ UTR.

Although the rice INF939 and SC938 libraries were generated from the same study and have similar read numbers [25], three motifs were identified in the INF939 library but no motifs were discovered in the SC938 library. During data processing, we noticed that many PARE ends from the SC938 library were terminated with “GC” dinucleotides. Therefore, we calculated the base composition of the last five nucleotides for all unique reads in the SC938 library and compared the result with that of the INF939 and NPBs libraries. We also calculated the base composition of rice cDNA for reference. The pattern of base composition was uniform among the last five nucleotides in the rice NPBs library and comparable to that of rice cDNA (Additional file 2: Figure S2). However, a dramatic distortion in base composition was seen in the last two nucleotides of all unique reads in the rice SC938 library and a mild distortion in the INF939 library. As the SC938 library was produced with the use of *Mme*I digestion which generates a 2-nt sticky end, selection bias might occur during the 3′-end ligation and thus distort the whole dataset.

We then searched the literature and databases for known motifs similar to the motif sequences we identified to reveal potential routes leading to small regulatory RNA-independent uncapped 5′-ends. Conservation of these motifs in different libraries or species other than Arabidopsis and rice was further examined by MORPH. Five motif groups that showed preferential accumulation of uncapped 5′-ends at the same position in Arabidopsis and rice and matched reported motifs or sequences are presented and discussed below.

### Presence of snoRNA 5′-ends in RNA degradome

**Figure 2:**
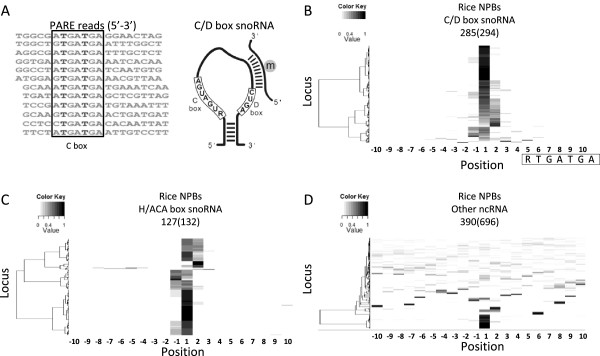
**The 5′-ends of rice snoRNAs were precisely captured in degradome data.** Motifs corresponding to the snoRNA C box are indicated in rice PARE reads and the schematic structure of a canonical C/D box snoRNA **(A)**. Uncapped reads produced around the 5′-ends of rice C/D box snoRNAs **(B)**, H/ACA box snoRNAs **(C)**, and other ncRNAs **(D)** identified previously are visualized by cluster heat maps. The first nucleotide of a snoRNA or an ncRNA was set to 1. The numbers of snoRNAs and ncRNAs reported previously (indicated in parentheses) and possessing uncapped reads in the region inspected are indicated above the heat map. Loci were clustered based on the distribution of normalized read numbers across the 20-nt region by Ward’s method.

snoRNAs are a class of non-coding RNAs (ncRNAs) that guide nucleotide modifications on rRNAs and snRNAs [32]. Most snoRNAs are abundant and either independently transcribed in the IGR or excised from the intron of polymerase-II-transcribed transcripts. Following transcription, the extra sequences in both termini of pre-snoRNAs are degraded by ribonucleases [33]. Consequently, mature snoRNAs usually lack a 5′ cap structure and a poly(A) tail. According to conserved motifs and RNA structures, snoRNAs are mainly divided into two groups, C/D box snoRNAs and H/ACA box snoRNAs, which direct methylation and pseudouridylation, respectively [32]. Besides sequence identity, several lines of evidence suggest that motif 1, RTGATGA (R = A or G), uncovered in the analysis is the C box of snoRNAs, and uncapped reads containing this motif, are likely derived from the 5′-end of snoRNAs: first, the motif was located at a precise position 5–6 nt downstream of the 5′-end of uncapped reads which is consistent with the location of a C box on snoRNAs (Figure 2A); second, this motif was mostly uncovered from the intron and IGR where snoRNAs are generally produced (Table 1); third, our previous study demonstrated that the 5′-end of known and novel Arabidopsis snoRNAs could be validated by PARE data [34]. Indeed, we found that uncapped 5′-ends carrying this motif largely overlapped with the 5′-end of known C/D box snoRNAs. We then reversely analyzed the proportion of rice snoRNA 5′ termini that could be precisely captured in the degradome. A cluster heat map was used to visualize the distribution of normalized uncapped reads around the 5′-ends for all known snoRNAs reported previously [35]. When setting the first nucleotide of snoRNAs to 1, almost all C/D box snoRNAs predominantly produced uncapped reads starting at position 1 or 1 nt deviated from 1 (Figure 2B). The conserved motifs of H/ACA box snoRNAs were not identified from the motif analysis because H and ACA boxes are located in the middle and the 3′-end of snoRNAs but not in the vicinity of snoRNA 5′-ends. However, uncapped reads could be also detected surrounding most H/ACA box snoRNA 5′ termini as observed in C/D box snoRNAs (Figure 2C). In contrast to snoRNAs, only a small fraction of other ncRNAs which were not annotated as snoRNAs had dominant accumulation of uncapped reads at the 5′-end (Figure 2D). In addition to the PARE dataset, datasets generated by degradome sequencing and the GMUCT method also contained Arabidopsis snoRNA 5′-ends, although to a lesser extent (Additional file 2: Figure S3). The comprehensive coverage of snoRNA 5′-ends in degradome data suggests that the degradome may alternatively be used in the validation of snoRNAs in addition to small RNA targets.

Mature and functional snoRNAs are 70–200 nt uncapped ncRNAs without a poly(A) tail and theoretically would not be captured by poly(T) beads which are used to enrich poly(A) RNA for deep sequencing. Unexpectedly, snoRNA 5′ termini were mostly and precisely found in Arabidopsis and rice PARE data but not the majority of other rice ncRNA 5′-ends. Variable 5′-ends of snoRNAs were also reported in the mouse degradome study [20]. A possible explanation for these unexpected results is that the snoRNAs detected by deep sequencing of uncapped 5′-ends might be polyadenylated intermediates instead of mature forms. Yeast exosome mutants show accumulation of 3′ extended polyadenylated snoRNAs which may represent intermediates during snoRNA maturation [36]. In contrast to polyadenylation on protein coding RNAs, which is a hallmark of mature transcripts, oligoadenylation on snoRNAs serves as a signal for 3′-to-5′ trimming in the exosome. A previous investigation of the 3′-end of poly(A) RNA in Arabidopsis by direct sequencing detected sequences downstream of snoRNA mature 3′ termini [37], supporting the existence of 3′ extended polyadenylated snoRNAs in wild-type plants. Since the PARE data used in this study only revealed the first 20 nt of uncapped RNA molecules from the 5′-end, it is not known whether plant snoRNAs captured in the degradome data have unprocessed 3′-ends like the snoRNA intermediates found in yeast exosome mutants. As the accuracy and throughput of sequencing transcripts longer than 200 nt have been much improved, a minor modification of the PARE protocol by replacing *Mme*I digestion with size fractionation for RNA species ranging 70–200 nt may provide a means to study these uncapped but polyadenylated snoRNAs.

### Association of uncapped 5′-ends with the PUF binding site

**Figure 3:**
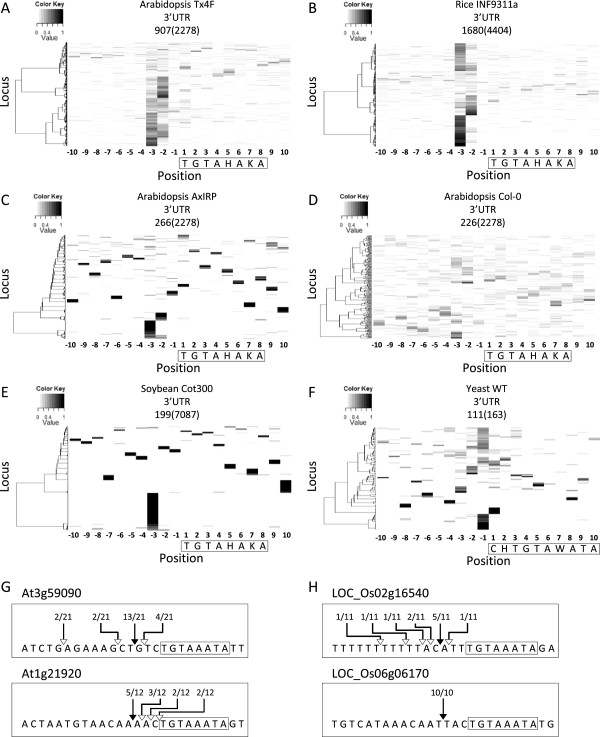
**Position-specific enrichment of uncapped 5′-ends surrounding putative PUF binding sites.** Distribution of normalized reads around putative PUF binding sites in the 3′ UTR of Arabidopsis genes with deep sequencing data derived from the PARE method **(A)**, degradome sequencing **(C)**, and GMUCT method **(D)** and rice **(B)**, soybean **(E)** and yeast **(F)** genes with PARE data. Motifs were boxed and the first nucleotide of motifs was set as 1. Loci containing the motif of interest were identified from the 3′ UTR of all annotated genes and the number is shown in parentheses above the heat map. For Arabidopsis and rice, only loci with a total read number greater than five in the 20-nt region are shown and the number of loci in each heat map is also indicated above the heat map. Loci were clustered based on the distribution of normalized read numbers across the 20-nt region by Ward’s method. Uncapped 5′-ends associated with putative PUF binding sites in Arabidopsis **(G)** and rice **(H)** were independently validated by the modified 5′ RACE protocol. The frequency of uncapped 5′-ends among clones sequenced at the position corresponding to the dominant termini supported by deep sequencing data is indicated with a filled arrow whereas at other positions it is indicated with an open arrow. Putative PUF binding sites are boxed.

Through a literature search, we found that motif 2, TGTAHAKA (H = A, T or C and K = T or G), is a highly conserved binding element of Pumilio/Fem-3 mRNA binding factor (PUF) proteins [38-41]. To exclude the possibility that the discovery of this motif is due to the frequent occurrences of the PUF binding site in the 3′ UTR of many genes, we examined the spatial relationship between the PUF binding site and uncapped reads on a genome-wide scale using MORPH. The genome-wide analysis showed prominent accumulation of uncapped reads at positions 2–3 nt upstream of the PUF binding site in all Arabidopsis and rice PARE datasets analyzed (Figure 3A, B and Additional file 2: Figure S4 and S5). On the other hand, when we shuffled the motif to ATTGAKAH, the enrichment of uncapped reads at the same position across libraries was no longer observed (Additional file 2: Figure S6 and S7). The increase of uncapped 5′-ends at positions 2–3 nt upstream of the PUF binding site was also observed in datasets generated by the degradome sequencing and GMUCT method but to a lesser extent (Figure 3C and D and Additional file 2: Figure S4). To further examine whether this is a common phenomenon across species, we then applied MORPH to soybean and budding yeast degradome datasets [18,19]. Although no reads were detected nearby the majority of putative PUF binding sites in the 3′ UTR of soybean genes, a bias in favor of the position 3-nt upstream of the PUF binding site was seen (Figure 3E). In the analysis of consensus motifs found in yeast PUF3, PUF4 and PUF5 targets [40], the position 1-nt upstream of the PUF3 consensus motif which is equivalent to the position 3-nt upstream of the plant PUF binding site also showed overrepresented uncapped 5′-ends (Figure 3F). The MORPH results indicated that the association of uncapped 5′-ends with PUF binding sites is highly conserved.

To rule out the possibility that these truncated transcripts appearing in degradome data were artifacts due to the high-throughput procedure, we selected six Arabidopsis and eight rice genes to validate the uncapped 5′-ends upstream of putative PUF binding sites by performing modified 5′ RACE individually. Although validation was not successful for every selected gene, we could clone 5′-ends located 2–3 nt upstream of putative PUF binding sites for two Arabidopsis genes and two rice genes (Figure 3G and H). The low success rate of modified 5′ RACE might be because the tissues or growth conditions we used were different from previous studies.

PUF proteins have been reported to be involved in mRNA decay through promoting deadenylation and in translational inhibition [42,43]. A recent study reported that human PUF binding sites are significantly enriched around miRNA target sites in the 3′ UTR and it has been demonstrated that PUF binding can induce RNA structural change that enhances miRNA accessibility in human cell lines [44,45]. Although PUF binding may enhance RNA decay through the miRNA pathway, many miRNAs in animals do not induce site-specific cleavage but promote deadenylation [46]. Moreover, most plant miRNAs target the CDS but not the 3′ UTR of transcripts and no miRNAs have been found in budding yeast, suggesting that uncapped 5′-ends specifically accumulated 2–3 nt upstream of the PUF binding site are unlikely to be the products of miRNA-guided cleavage. Taken together, PUF binding may result in the production of uncapped 5′-ends through an uncharacterized but common mechanism.

### Association of uncapped 5′-ends with a poly(A) signal-like element

**Figure 4:**
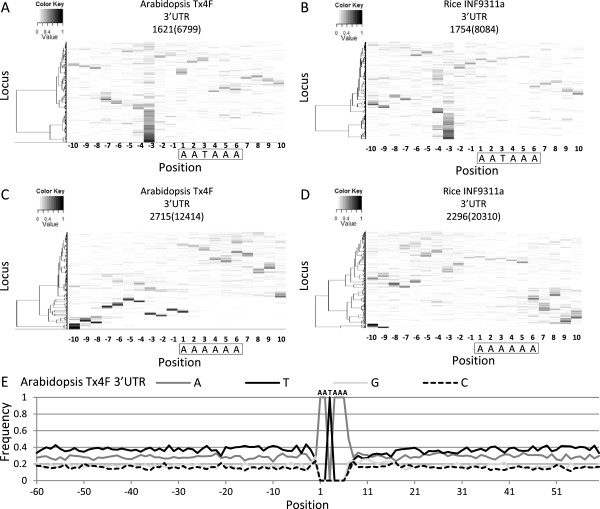
**Position-specific enrichment of uncapped 5′-ends surrounding a poly(A) signal-like element.** Distribution of normalized reads around the AATAAA and AAAAAA sites in the 3′ UTR of Arabidopsis genes **(A and C)** and rice genes **(B and D)** is visualized by MORPH. Loci containing the motif of interest were identified from the 3′ UTR of all annotated genes and the number is shown in parentheses above the heat map. Only loci with a total read number greater than five in the 20-nt region are shown and the number of loci in each heat map is also indicated above the heat map. Motifs were boxed with the first nucleotide set as 1. Loci were clustered based on the distribution of normalized read numbers across the 20-nt region by Ward’s method. Base composition upstream and downstream of the AATAAA sites identified in the motif analysis of Tx4F library in the 3′ UTR of Arabidopsis genes **(E)**.

An adenosine rich motif AATAAA, motif 3, was revealed in the Arabidopsis 3′ UTR (Table 1). When performing a genome-wide analysis to explore the association between AATAAA and uncapped reads using MORPH, a dominant occurrence of uncapped reads at a position 3 nt upstream of AATAAA sites could be observed in all the Arabidopsis and rice PARE libraries analyzed except the rice SC938 library (Figure 4A and B and Additional file 2: Figure S8). When modifying the motif to AAAAAA, the preferential accumulation of PARE reads at this position was abolished (Figure 4C and D). The specific and conserved distance between AATAAA and the 5′-end of uncapped reads across libraries and two species suggests that the discovery of this motif is not due to the over-representation of AATAAA in plant 3′ UTR. AATAAA is a universal signal for polyadenylation in animals [47]. However, less than 20% of Arabidopsis genes possess AATAAA in the proximity of the polyadenylation site [37]. We further compared the properties of these AATAAA sites with those of the canonical poly(A) signal. First, the average distance of these AATAAA sites identified from the analysis of the Tx4F and INF9311a libraries to the 3′ terminus of genes was 131 and 227 nt while the canonical poly(A) signal is usually located 10–30 nt upstream of the polyadenylation site [48]. Second, the base composition of 60-nt regions upstream and downstream of these AATAAA sites was comparable while a U-rich region was frequently found downstream of the canonical poly(A) signal in Arabidopsis (Figure 4E) [37]. Therefore, AATAAA identified in our study may not function as a canonical poly(A) signal. The canonical poly(A) signal guides cleavage and polyadenylation by recruiting cleavage/polyadenylation specificity factors (CPSFs) [49,50]. The sequence homology suggests that this poly(A) signal-like motif might be recognized by proteins possessing similar RNA binding domains of CPSFs. However, the function of this poly(A) signal-like element in RNA processing or degradation remains to be elucidated.

### Association of uncapped 5′-ends with RNA binding motifs

**Figure 5:**
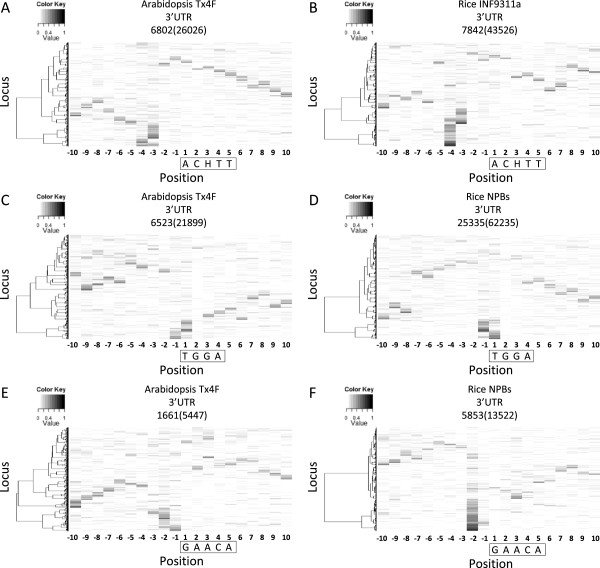
**Position-specific enrichment of uncapped 5′-ends surrounding three motifs recognized by RNA-binding proteins.** Distribution of normalized reads around three motifs recognized by RNA-binding proteins in the 3′ UTR of Arabidopsis genes **(A , C and E)** and rice genes **(B , D and F)** is visualized by MORPH. ACHTT and TGGA are motifs bound by *Physcomitrella patens* Phpat.016g078400 (Pp_0206) and Phpat.012g050300 (Pp_0237) respectively. GAACA is a motif bound by *Ostreococcus tauri* Ot09g01160 (Ot_0263). Loci containing the motif of interest were identified from the 3′ UTR of all annotated genes and the number is shown in parentheses above the heat map. Only loci with a total read number greater than five in the 20-nt region are shown and the number of loci in each heat map is also indicated. Motifs were boxed with the first nucleotide set as 1. Loci were clustered based on the distribution of normalized read numbers across the 20-nt region by Ward’s method.

The identification of the PUF binding site and a poly(A) signal-like element associated with the production of uncapped 5′-ends at specific positions across species raises the question of whether motifs recognized by other RNA binding proteins might show similar phenomena. To answer this question, we used MORPH to examine the distribution of uncapped 5′-ends surrounding seven motifs which were reported to be recognized by plant RNA binding proteins [51]. Three of them showed position-specific enrichment of uncapped 5′-ends immediately or a few nucleotides upstream of the motifs (Figure 5). Notably, the enrichment occurred at the same or close positions among different Arabidopsis and rice PARE libraries (Figure 5 and Additional file 2: Figure S9, S10, and S11). The result suggests a possible connection between protein binding and production of uncapped 5′-ends in the nearby region.

Although specifically truncated termini are commonly the result of endonucleolytic cleavage, stalling of exoribonuclease trimming can also generate precise termini during RNA maturation. For instance, maturation of snoRNA 5′-ends in the nucleus requires trimming precursors with 5′-to-3′ exoribonucleases [7]. The protein binding to conserved snoRNA motifs delineates mature 5′ termini by preventing exoribonuclease processing. Resembling the proteins associated with snoRNAs, plant pentatricopeptide repeat (PPR) proteins bound to chloroplast RNA termini are thought to impede 5′ and 3′ degradation and thus serve as the determinants of chloroplast RNA maturation [52,53]. Interestingly, small RNAs overlapping PPR binding sites on chloroplast RNAs have been reported in both monocots and dicots [53,54]. Similarly, small RNAs were enriched at the snoRNA 5′-end in animals and plants [34,55]. These small RNAs may represent the footprints of RNA binding proteins. Although the formation of nuclear-encoded mRNA 5′-ends generally does not require exoribonucleotlytic trimming, we suspect that when mRNAs are decapped and subjected to degradation by 5′-to-3′ exoribonucleases, the region occupied by RNA binding proteins may be less accessible to exoribonucleases and thus form a relatively stable and defined terminus. Therefore, our results may imply that RNA degradome data contain the footprints of various RNA binding proteins.

### Association of uncapped 5′-ends with a CAGAC motif in the 3′ UTR

**Figure 6:**
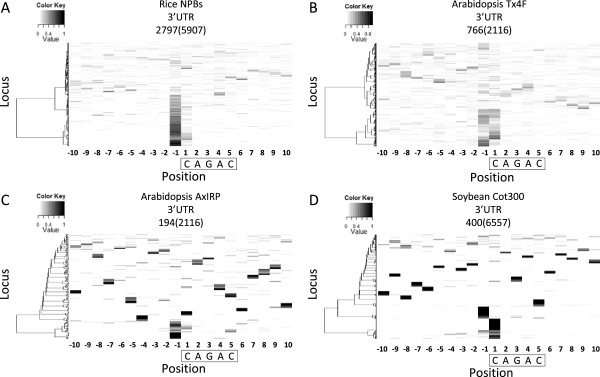
**Position-specific enrichment of uncapped 5′-ends surrounding a CAGAC motif in the 3′ UTR.** Distribution of normalized reads around a CAGAC motif in the 3′ UTR of rice genes **(A)**, Arabidopsis genes **(B and C)** and soybean genes **(D)** is visualized by MORPH. Loci containing the motif of interest were identified from the 3′ UTR of all annotated genes and the number is shown in parentheses above the heat map. For rice and Arabidopsis, only loci with a total read number greater than five in the 20-nt region are shown and the number of loci in each heat map is also indicated. The motif was boxed with the first nucleotide set as 1. Loci were clustered based on the distribution of normalized read numbers across the 20-nt region by Ward’s method.

Although motif 7, CAGAC, was only identified in the rice NPBs library (Table 1), the other three rice and two Arabidopsis PARE libraries also showed more accumulation of uncapped 5′-ends at the position immediately or 1-nt upstream of this motif compared to other positions in the 3′ UTR (Figure 6A, B and Additional file 2: Figure S12). Enrichment of uncapped 5′-ends at the same position around this motif was also seen in Arabidopsis AxIRP library generated by degradome sequencing although to a much lesser extent (Figure 6C). Moreover, uncapped 5′-ends produced in the proximity of this motif in the 3′ UTR of soybean genes tended to be overrepresented at the same position (Figure 6D). Motif 7 is highly similar to the Smad binding element (SBE) found in the promoter region of transforming growth factor β (TGFβ) target genes in metazoan [56]. Recently, the binding of Smad proteins to CAGAC on the stem of pri-miRNA has been shown to promote miRNA maturation by facilitating the recruitment of Drosha [57]. Although the TGFβ/Smad signaling pathway is absent in the Arabidopsis genome [58], the association of CAGAC with uncapped 5′-ends in the 3′ UTR raises the possibility that this motif in plants may be bound by a Smad-like protein and trigger post-transcriptional regulation of mRNA analogous to the regulation of pri-miRNA by Smad proteins in humans. The uncapped 5′-ends associated with this motif might thus also be the footprint of proteins bound to CAGAC.

### Sequencing artifacts resulting from non-specific PCR amplification

**Figure 7:**
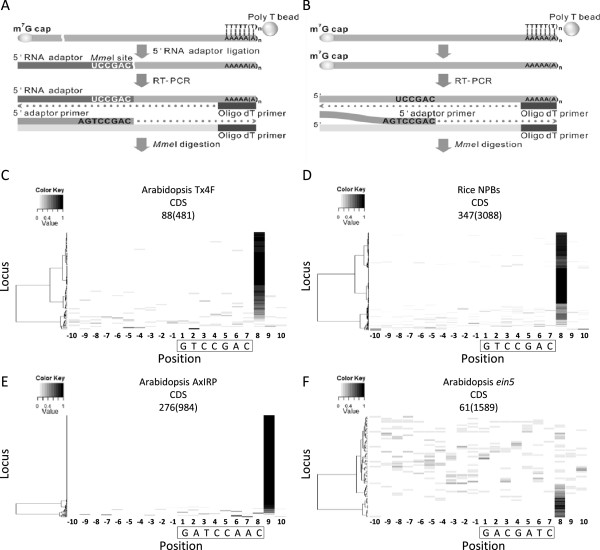
**Potential false uncapped 5′-ends caused by non-specific PCR amplification.** Schemas depict models of uncapped transcripts **(A)** and capped transcripts **(B)** captured by the PARE protocol. The 5′ adaptor primer was perfectly annealed to cDNA corresponding to the 5′ RNA adaptor ligated to uncapped transcripts whereas it was partially annealed at its 3′-end to the internal region of capped cDNA. Distribution of normalized uncapped reads around the GTCCGAC sites in the CDS of Arabidopsis **(C)** and rice **(D)** genes with PARE libraries is visualized by MORPH. Reads surrounding motifs corresponding to the 3′ end of the 5′ adaptors used in degradome sequencing **(E)** and GMUCT **(F)** method are also visualized by MORPH. Motifs were boxed with the first nucleotide set as 1. Loci containing the motif of interest were identified from the CDS of all annotated genes and the number is shown in parentheses above the heat map. Only loci with a total read number greater than five in the 20-nt region are shown and the number of loci in each heat map is also indicated. Motifs were boxed with the first nucleotide set as 1. Loci were clustered based on the distribution of normalized read numbers across the 20-nt region by Ward’s method.

Motifs 9, 10, and 11 all occurred immediately upstream of uncapped 5′-ends and both motifs 9 and 10 had a *Mme*I site (CCRAC; R = A or G) at the 3′-end (Table 1). To our surprise, the sequence of motif 9 matched the 3′ terminal sequence of the 5′ adaptor primer used in PARE library construction. Considering the sequence identity and the unique location of this motif, we speculated that this motif might represent an artifact of uncapped 5′-ends produced during PARE library construction. In the PARE protocol, a 5′ adaptor primer containing AGTCCGAC at its most 3′-end was used to amplify cDNA before *Mme*I digestion for subsequent sequencing (Figure 7A) [59]. Some capped transcripts possessing internal sequences which could anneal with the 5′ adaptor primer especially at the 3′-end might be converted into cDNA although they were not ligated to a 5′ RNA adaptor (Figure 7B). To further examine this artifact on a genome-wide scale, we adopted MORPH to visualize the occurrences of PARE reads surrounding GTCCGAC sites. Strikingly, almost all loci with reads over five around this motif in the CDS showed an obvious increase of PARE reads at a position immediately downstream of GTCCGAC sites compared to that at other 19 positions for Arabidopsis Tx4f and rice NPBs libraries (Figure 7C and D). Therefore, these *Mme*I-site-associated PARE reads might be derived from intact mRNAs with a 5′ cap but were amplified through non-specific annealing of the 5′ adaptor primer.

Interestingly, the motif analysis of the AxIDT, AxIRP, and AxSRP libraries generated by the degradome sequencing with the use of *Mme*I digestion also revealed an *Mme*I-site containing motif (motif 10) at the same position but with minor sequence difference (Table 1). Strong enrichment of uncapped 5′-ends immediately downstream of motif 10 could be also observed on the genome-wide scale (Figure 7E). The minor sequence difference between motifs 9 and 10 could be explained by the different 5′ adaptor primers used in library construction for the PARE protocol and degradaome sequencing. For the GMUCT libraries (Col-0 and *ein5*) which were constructed through sonication instead of enzyme digestion, *Mme*I-site containing motifs were not recovered by MEME analysis whereas a distinct motif, motif 11, corresponding to the 3′-end sequence of the 5′ RNA adaptor used in the GMUCT method was found at the same position (Table 1) [15]. The enrichment of uncapped 5′-ends immediately downstream of motif 11 was seen but less evident in the GMUCT libraries on a genome-wide scale (Figure 7F). Unlike the PARE method and degradome sequencing, the 3′ terminus of the GMUCT 5′ adaptor primer was a few nucleotides upstream of the 3′ terminus of the 5′ RNA adaptor which ligates to the uncapped 5′-end. This arrangement could help eliminate the artifact of non-specific PCR amplification during the trimming of 5′ adaptor sequence. In summary, these three upstream motifs suggest that non-specific PCR amplification could occur in genome-wide analysis of uncapped ends regardless of the use of enzyme digestion or sonication. This result raises some concern about the presence of this artifact in public genome-wide data of uncapped 5′-ends.

## Conclusions

Deep sequencing of uncapped 5′-ends provides an unprecedented opportunity to investigate transient and stable RNA intermediates produced during RNA processing and RNA turnover at the level of the genome. As RNA silencing represents one of many pathways involved in RNA degradation, bioinformatics analysis from a perspective independent of small RNA-guided cleavage is crucial for detailed understanding of degradome data. The motif analysis performed in this study provides clues about the significant but overlooked RNA population in degradome data. Polyadenylated ncRNAs, potential footprints of RNA binding proteins and artifacts derived from non-specific PCR amplification may all contribute to the complexity of RNA degradome data. These findings increase our understanding of RNA species that can be captured by deep sequencing of uncapped 5′-ends and may lead to alternative applications of degradome data in the study of ncRNA processing and the identification of target sites for RNA binding proteins.

## Materials and Methods

### Sequence data

The genes, genomic sequences and degradome datasets used in this study were downloaded from the following public databases. Two Arabidopsis PARE libraries, three Arabidopsis degradome sequencing libraries, two Arabidopsis GMUCT libraries, four rice PARE libraries, one soybean PARE library and one yeast PARE library were retrieved from the Gene Expression Omnibus (GEO, http://www.ncbi.nlm.nih.gov/geo/) [13-15,18,19,21,23,25]. The accession numbers of 13 libraries are listed in Additional file 1: Table S2. For PARE libraries, only 20-nt reads were used in mapping and subsequent analyses while the first 20 nt of reads were used for GMUCT libraries. Reference sequences and the annotation of Arabidopsis and rice genomes used in mapping uncapped reads were downloaded from TAIR (http://www.arabidopsis.org/, TAIR 10) and MSU Rice Genome Annotation (http://rice.plantbiology.msu.edu/, Release 6.1). Rice snoRNAs and putative intermediate-sized ncRNAs were collected from the report of Liu et al. [35]. Known Arabidopsis and rice miRNA targets previously used to evaluate the performance of the SeqTar method were adopted in this study [28]. Yeast genome sequence was downloaded from Saccharomyces Genome Database (http://www.yeastgenome.org/) and the sequences of yeast 3′ UTR were based on the annotation used in the previous yeast PARE study [19]. Soybean genome sequences and annotation were retrieved from phytozome (http://www.phytozome.net/soybean.php).

### Motif analysis

To discover position-specific motifs associated with predominant uncapped 5′-ends in each genomic region, the standalone MEME suite was used in the analysis of 50-nt sequences (25-nt upstream and 25-nt downstream) flanking selected uncapped 5′-ends with the following parameters: 6–8 nt motifs which occur zero or once in the given strand per input sequence and each motif must occur at least at five sites [30].

### Motif-oriented read positioning heat map (MORPH)

Cluster analysis and heat map graphing were carried out with R statistical software (http://www.r-project.org/) to visualize the distribution of normalized uncapped reads surrounding motifs on a genome-wide scale. The position of an uncapped read was defined by its 5′ terminus relative to the first nucleotide of motifs which was set as 1. Positions upstream of motifs were indicated by negative values while downstream positions were indicated by positive values. Uncapped reads occurring within a 20-nt region flanking every motif site found in a genomic region were extracted. Next, the read number at each position was normalized by the total reads occurring within the 20-nt region for each locus. Finally, loci were clustered based on the distribution of normalized read numbers across the 20-nt region by Ward’s method with R package.

### Plant materials and RNA isolation

Rice (*Oryza sativa* ssp. *japonica* cv. Tainung 67) was hydroponically cultured in half-strength Kimura B nutrient medium under a 16/8-h light/dark period and 30/28°C day/night temperature. *Arabidopsis thaliana* (ecotype Col-0) used in this study was grown on 0.8% Bacto-agar plates containing half-strength MS and 1% sucrose under a 16/8-h light/dark cycle at 22°C. Total RNA of 7-day-old Arabidopsis seedlings and 2-week-old rice seedlings were extracted with Plant RNA Purification Reagent (Invitrogen) and MaxTract high-density gel tubes (Qiagen) for the modified 5′ RACE assay.

### Modified 5′ RACE assay

Modified 5′ RACE assay was performed to validate uncapped 5′-ends using GeneRacer Kit (Invitrogen). First, poly(A) RNA purified from 50–100 μg total RNA using the MicroPoly(A) Purist Kit (Ambion) was ligated with the 5′ RNA adapter and reversely transcribed with the oligo-dT primer. cDNA was used as template for nested PCR analysis. The primary PCR was performed using the GeneRacer 5′ primer and a gene-specific primer, followed by secondary PCR using the GeneRacer 5′ nested primer with a gene-specific nested primer. Amplified products of expected size were gel purified, cloned into pJET1.2/blunt cloning vector (Thermo) and sequenced. The primers used in this study are listed in Additional file 1: Table S3.

## Competing interests

The authors declared that they have no competing interests.

## Authors’ contributions

CYH, MTW, SHL and HMC analyzed degradome data. CYH and MTW carried out modified 5′ RACE assay. CYH, MTW, YIH and HMC wrote the manuscript. All authors read and approved the final manuscript.

## Supplementary Material

Additional file 1: Table S1-S3 **Table S1.** The numbers of uncapped 5′-ends passing the statistical test, corresponding to cleavage sites guided by miRNAs and used in MEME analysis for different libraries in distinct genomic regions. **Table S2.** The information of degradome libraries used in this study. **Table S3.** List of primers used in modified 5′ RACE analysis. Click here for file

Additional file 2: Figure S1-S12 **Figure S1.** A CA-repeat associated with uncapped 5′-ends in the 3′ UTR and CDS of rice genes. **Figure S2.** Bias of base composition in the 3′-end of rice SC938 degradome reads. **Figure S3.** The 5′-ends of Arabidopsis snoRNAs captured by three sequencing approaches. **Figure S4.** Position-specific enrichment of uncapped 5′-ends surrounding putative PUF binding sites across Arabidopsis degradome libraries. **Figure S5.** Position-specific enrichment of uncapped 5′-ends surrounding putative PUF binding sites across rice PARE libraries. **Figure S6.** Distribution of uncapped 5′-ends surrounding a shuffled PUF motif for Arabidopsis degradome libraries. **Figure S7.** Distribution of uncapped 5′-ends surrounding a shuffled PUF motif for rice degradome libraries. **Figure S8.** Position-specific enrichment of uncapped 5′-ends surrounding a poly(A) signal-like element across PARE libraries and species. **Figure S9.** Position-specific enrichment of uncapped 5′-ends surrounding an ACHTT motif across PARE libraries and species. **Figure S10.** Position-specific enrichment of uncapped 5′-ends surrounding a TGGA motif across PARE libraries and species. **Figure S11.** Position-specific enrichment of uncapped 5′-ends surrounding a GAACA motif across PARE libraries and species. **Figure S12.** Position-specific enrichment of uncapped 5′-ends surrounding a CAGAC motif across PARE libraries and species. Click here for file
