# Supplementary material for: Beyond cleaved small RNA targets: unraveling the complexity of plant RNA degradome data
Source: BMC Genomics. 2014 Jan 10;15:15. doi: 10.1186/1471-2164-15-15 (PMC3898255; doi:10.1186/1471-2164-15-15)
Supplement: Additional file 1: Table S1-S3 — Table S1. The numbers of uncapped 5′-ends passing the statistical test, corresponding to cleavage sites guided by miRNAs and used in MEME analysis for different libraries in distinct genomic regions. Table S2. The information of degradome libraries used in this study. Table S3. List of primers used in modified 5′ RACE analysis. [file 1471-2164-15-15-S1.docx]

**Table S1.** **The numbers of uncapped 5**′**-ends passing the statistical test, corresponding to cleavage sites guided by miRNAs, and used in MEME analysis for different libraries in distinct genomic regions**

| **Library** | **Region^a^** | **Binomial^b^** | **miRNA targets filtered^c^** | | **MEME^d^** |
| --- | --- | --- | --- | --- | --- |
| AxIDT | 3'UTR | 744 | 2 | 742 | |
| AxIRP |  | 21293 | 3 | 1000 | |
| AxSRP |  | 17845 | 2 | 1000 | |
| Col-0 |  | 468 | 2 | 466 | |
| *ein5* |  | 436 | 3 | 433 | |
| TWF |  | 26341 | 13 | 1000 | |
| Tx4F |  | 4434 | 14 | 1000 | |
| AxIDT | 5'UTR | 9 | 0 | 9 | |
| AxIRP |  | 2255 | 2 | 1000 | |
| AxSRP |  | 1317 | 0 | 1000 | |
| Col-0 |  | 26 | 0 | 26 | |
| *ein5* |  | 41 | 0 | 41 | |
| TWF |  | 6875 | 3 | 1000 | |
| Tx4F |  | 3480 | 4 | 1000 | |
| AxIDT | CDS | 383 | 3 | 380 | |
| AxIRP |  | 47566 | 23 | 1000 | |
| AxSRP |  | 25891 | 20 | 1000 | |
| Col-0 |  | 441 | 3 | 438 | |
| *ein5* |  | 422 | 8 | 414 | |
| TWF |  | 112319 | 88 | 1000 | |
| Tx4F |  | 10375 | 91 | 1000 | |
| AxIDT | IGR | 100 | 0 | 100 | |
| AxIRP |  | 3465 | 0 | 1000 | |
| AxSRP |  | 1795 | 0 | 1000 | |
| Col-0 |  | 217 | 0 | 217 | |
| *ein5* |  | 308 | 0 | 308 | |
| TWF |  | 8944 | 0 | 1000 | |
| Tx4F |  | 2689 | 0 | 1000 | |
| AxIDT | intron | 32 | 0 | 32 | |
| AxIRP |  | 1885 | 0 | 1000 | |
| AxSRP |  | 1092 | 0 | 1000 | |
| Col-0 |  | 44 | 0 | 44 | |
| *ein5* |  | 63 | 0 | 63 | |
| TWF |  | 5279 | 1 | 1000 | |
| Tx4F |  | 729 | 0 | 729 | |
| INF9311a | 3'UTR | 8188 | 9 | 1000 | |
| INF939 |  | 2651 | 6 | 1000 | |
| NPBs |  | 21828 | 23 | 1000 | |
| SC938 |  | 26845 | 5 | 1000 | |
| INF9311a | 5'UTR | 123 | 0 | 123 | |
| INF939 |  | 57 | 0 | 57 | |
| NPBs |  | 923 | 3 | 920 | |
| SC938 |  | 98 | 0 | 98 | |
| INF9311a | CDS | 1878 | 28 | 1000 | |
| INF939 |  | 554 | 10 | 544 | |
| NPBs |  | 8309 | 55 | 1000 | |
| SC938 |  | 1211 | 2 | 1000 | |
| INF9311a | IGR | 5079 | 0 | 1000 | |
| INF939 |  | 64 | 0 | 64 | |
| NPBs |  | 6155 | 0 | 1000 | |
| SC938 |  | 550 | 0 | 550 | |
| INF9311a | intron | 800 | 0 | 800 | |
| INF939 |  | 81 | 0 | 81 | |
| NPBs |  | 2414 | 2 | 1000 | |
| SC938 |  | 648 | 0 | 648 | |

^a^IGR, UTR and CDS indicate the intergenic region, the untranslated region and the coding sequence, respectively

^b^The number of unique uncapped 5′-ends passing the binomial test with a cutoff of *P*-value <10^-5^

^c^The number of unique uncapped 5′-ends corresponding to the cleavage sites initiated by Arabidopsis and rice miRNAs and passing the binomial test with a cutoff of *P*-value <10^-5^. These uncapped 5′-ends were filtered and not included in the subsequent motif analysis with the MEME suite

^d^The number of unique ends used in the motif analysis

**Table S2. The information of degradome libraries used in this study**

| **Species** | **GEO^a^** | **Code^b^** | **Read^c^** | **Approach^d^** |
| --- | --- | --- | --- | --- |
| Arabidopsis | GSM278334 | AxIDT | 1,090,514 | Degradome sequencing |
|  | GSM278335 | AxIRP | 3,553,023 |  |
|  | GSM278370 | AxSRP | 3,117,154 |  |
|  | GSM284751 | Col-0 | 3,410,152 | GMUCT |
|  | GSM284752 | *ein5* | 6,335,580 |  |
|  | GSM280226 | TWF | 17,711,729 | PARE |
|  | GSM280227 | Tx4F | 10,643,828 |  |
| Rice | GSM455938 | SC938 | 4,494,395 | PARE |
|  | GSM455939 | INF939 | 4,426,044 |  |
|  | GSM476257 | INF9311a | 12,314,353 |  |
|  | GSM434596 | NPBs | 39,376,422 |  |
| Soybean | GSM848967 | Cot300 | 41,517,379 |  |
| Yeast | GSM940394 | WT | 142,023,128 |  |

^a^The accession number at NCBI's Gene Expression Omnibus (GEO).

^b^The library name.

^c^The total read number of each library.

^d^Sequencing approaches for genome-wide analysis of uncapped 5’-ends; genome-wide mapping of uncapped transcripts (GMUCT) and parallel analysis of RNA ends (PARE).

**Table S3. List of primers used in modified 5' RACE analysis**

| **Primer** | **Sequence** |
| --- | --- |
| GeneRacer 5' | CGACTGGAGCACGAGGACACTGA |
| GeneRacer 5'_nested | GGACACTGACATGGACTGAAGGAGTA |
| At3g59090 3' | AAAACCAGAATGATGCGTCGTCGTATATGTAAATG |
| At3g59090 3'_nested | TATATGTAAATGATAACAAAGCAGCTTAAACGACGC |
| At1g21920 3' | GATCAAAACCAAAATAAGCTCCATTTCAAGCC |
| LOC_Os02g16540 3' | GGATTTTTTTTTGCCATTAGAGGGAGCTTCTTTCTC |
| LOC_Os02g16540 3'_nested | CTTCTTTCTCTCTTCTTCAAGCTTACAAACATCCC |
| LOC_Os06g06170 3' | CATGTCTTTGCAGCCGCGG |
| LOC_Os06g06170 3'_nested | CCGCGGCTGCAGGTCC |
