# Supplementary material for: Beyond cleaved small RNA targets: unraveling the complexity of plant RNA degradome data
Source: BMC Genomics. 2014 Jan 10;15:15. doi: 10.1186/1471-2164-15-15 (PMC3898255; doi:10.1186/1471-2164-15-15)
Supplement: Additional file 2: Figure S1-S12 — Figure S1. A CA-repeat associated with uncapped 5′-ends in the 3′ UTR and CDS of rice genes. Figure S2. Bias of base composition in the 3′-end of rice SC938 degradome reads. Figure S3. The 5′-ends of Arabidopsis snoRNAs captured by three sequencing approaches. Figure S4. Position-specific enrichment of uncapped 5′-ends surrounding putative PUF binding sites across Arabidopsis degradome libraries. Figure S5. Position-specific enrichment of uncapped 5′-ends surrounding putative PUF binding sites across rice PARE libraries. Figure S6. Distribution of uncapped 5′-ends surrounding a shuffled PUF motif for Arabidopsis degradome libraries. Figure S7. Distribution of uncapped 5′-ends surrounding a shuffled PUF motif for rice degradome libraries. Figure S8. Position-specific enrichment of uncapped 5′-ends surrounding a poly(A) signal-like element across PARE libraries and species. Figure S9. Position-specific enrichment of uncapped 5′-ends surrounding an ACHTT motif across PARE libraries and species. Figure S10. Position-specific enrichment of uncapped 5′-ends surrounding a TGGA motif across PARE libraries and species. Figure S11. Position-specific enrichment of uncapped 5′-ends surrounding a GAACA motif across PARE libraries and species. Figure S12. Position-specific enrichment of uncapped 5′-ends surrounding a CAGAC motif across PARE libraries and species. [file 1471-2164-15-15-S2.pptx]

## Slide 1
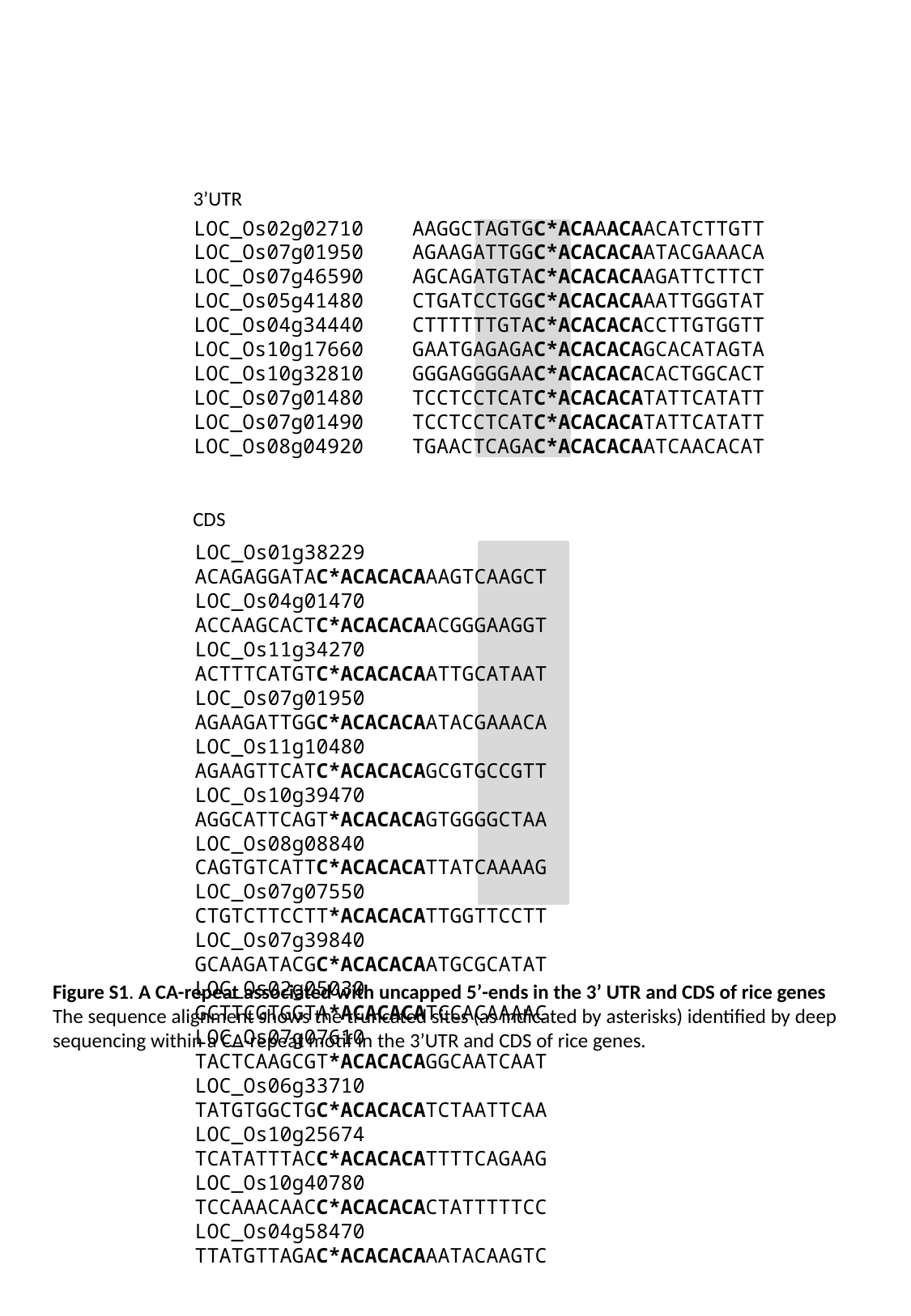

3’UTR
LOC_Os02g02710 AAGGCTAGTGC*ACAAACAACATCTTGTT
LOC_Os07g01950 AGAAGATTGGC*ACACACAATACGAAACA
LOC_Os07g46590 AGCAGATGTAC*ACACACAAGATTCTTCT
LOC_Os05g41480 CTGATCCTGGC*ACACACAAATTGGGTAT
LOC_Os04g34440 CTTTTTTGTAC*ACACACACCTTGTGGTT
LOC_Os10g17660 GAATGAGAGAC*ACACACAGCACATAGTA
LOC_Os10g32810 GGGAGGGGAAC*ACACACACACTGGCACT
LOC_Os07g01480 TCCTCCTCATC*ACACACATATTCATATT
LOC_Os07g01490 TCCTCCTCATC*ACACACATATTCATATT
LOC_Os08g04920 TGAACTCAGAC*ACACACAATCAACACAT
CDS
LOC_Os01g38229 ACAGAGGATAC*ACACACAAAGTCAAGCT
LOC_Os04g01470 ACCAAGCACTC*ACACACAACGGGAAGGT
LOC_Os11g34270 ACTTTCATGTC*ACACACAATTGCATAAT
LOC_Os07g01950 AGAAGATTGGC*ACACACAATACGAAACA
LOC_Os11g10480 AGAAGTTCATC*ACACACAGCGTGCCGTT
LOC_Os10g39470 AGGCATTCAGT*ACACACAGTGGGGCTAA
LOC_Os08g08840 CAGTGTCATTC*ACACACATTATCAAAAG
LOC_Os07g07550 CTGTCTTCCTT*ACACACATTGGTTCCTT
LOC_Os07g39840 GCAAGATACGC*ACACACAATGCGCATAT
LOC_Os02g05030 GCTTCGTGGTA*ACACACATCCACAAAAC
LOC_Os07g07610 TACTCAAGCGT*ACACACAGGCAATCAAT
LOC_Os06g33710 TATGTGGCTGC*ACACACATCTAATTCAA
LOC_Os10g25674 TCATATTTACC*ACACACATTTTCAGAAG
LOC_Os10g40780 TCCAAACAACC*ACACACACTATTTTTCC
LOC_Os04g58470 TTATGTTAGAC*ACACACAAATACAAGTC
Figure S1. A CA-repeat associated with uncapped 5’-ends in the 3’ UTR and CDS of rice genes
The sequence alignment shows the truncated sites (as indicated by asterisks) identified by deep sequencing within a CA-repeat motif in the 3’UTR and CDS of rice genes.

## Slide 2
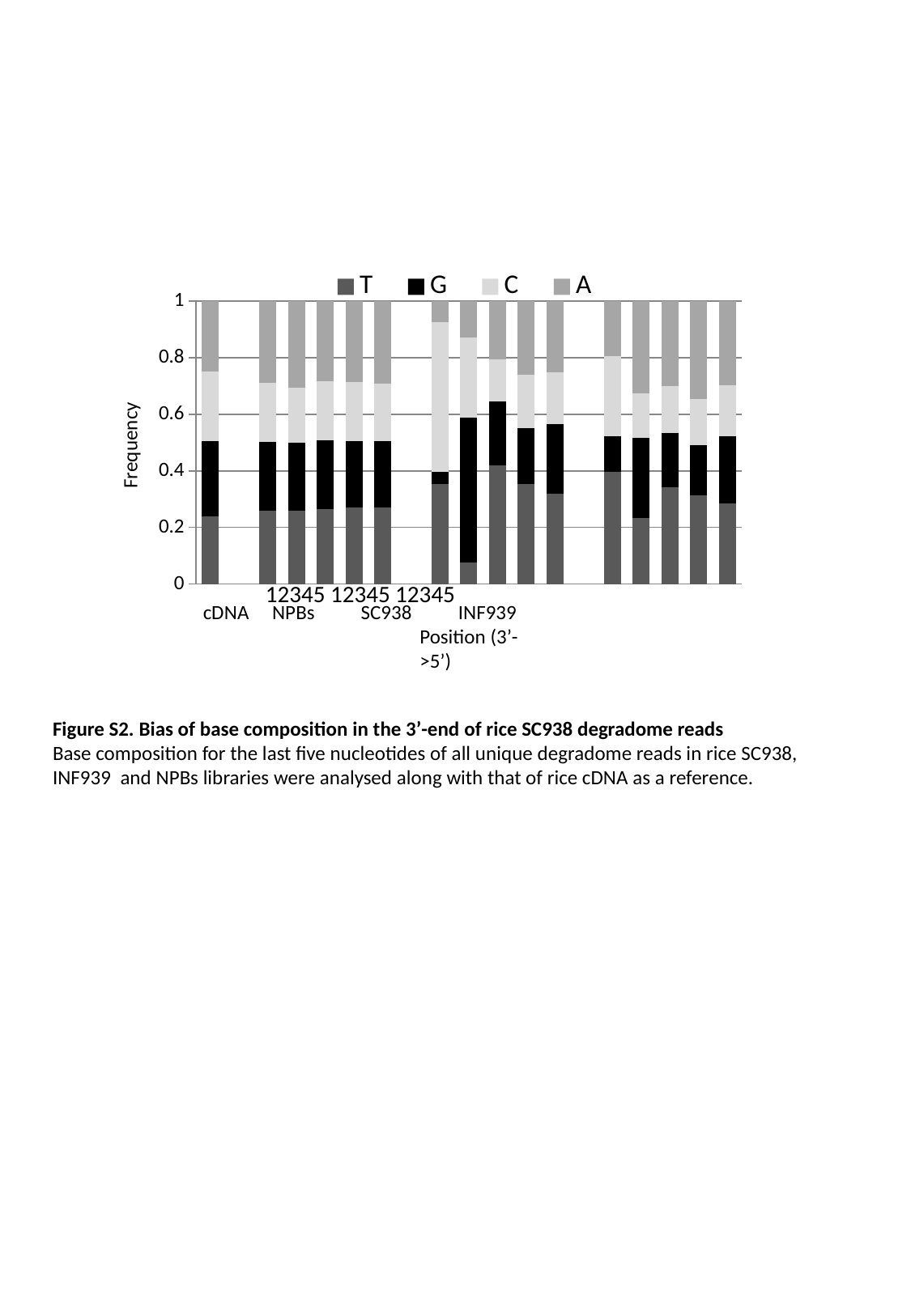

### Chart
| Category | T | G | C | A |
|---|---|---|---|---|
| rice_cDNA | 23.82379017 | 26.58043744 | 24.66337784 | 24.87218714 |
| | None | None | None | None |
| 1st | 25.91083503 | 24.30728964 | 20.83761324 | 28.83334587 |
| 2nd | 25.89145494 | 23.88553492 | 19.43972974 | 30.682601 |
| NPBs 3rd | 26.51502141 | 24.07934875 | 21.04690525 | 28.26250359 |
| 4th | 27.1472523 | 23.18984476 | 20.93818263 | 28.63396714 |
| 5th | 27.11142988 | 23.24762266 | 20.45565402 | 29.10596773 |
| | None | None | None | None |
| 1st | 35.38289019 | 4.2256216 | 53.02722785 | 7.364260353 |
| 2nd | 7.694254588 | 51.07668945 | 28.26760898 | 12.96144699 |
| SC938 3rd | 41.79616165 | 22.84105056 | 14.88171069 | 20.4810771 |
| 4th | 35.49224241 | 19.47813682 | 18.98696008 | 26.04266069 |
| 5th | 31.97153694 | 24.59165484 | 18.33968698 | 25.09712124 |
| | None | None | None | None |
| 1st | 39.64102505 | 12.49495614 | 28.36980543 | 19.49421338 |
| 2nd | 23.48511668 | 28.17160329 | 15.80021417 | 32.54306586 |
| INF939 4th | 34.09495742 | 19.15033308 | 16.61605182 | 30.13865768 |
| 4th | 31.4896594 | 17.55135237 | 16.25513742 | 34.70385081 |
| 5th | 28.46619249 | 23.62628481 | 18.07435015 | 29.83317256 |Frequency
12345 12345 12345
 cDNA NPBs SC938 INF939
Position (3’->5’)
Figure S2. Bias of base composition in the 3’-end of rice SC938 degradome reads
Base composition for the last five nucleotides of all unique degradome reads in rice SC938, INF939 and NPBs libraries were analysed along with that of rice cDNA as a reference.

## Slide 3
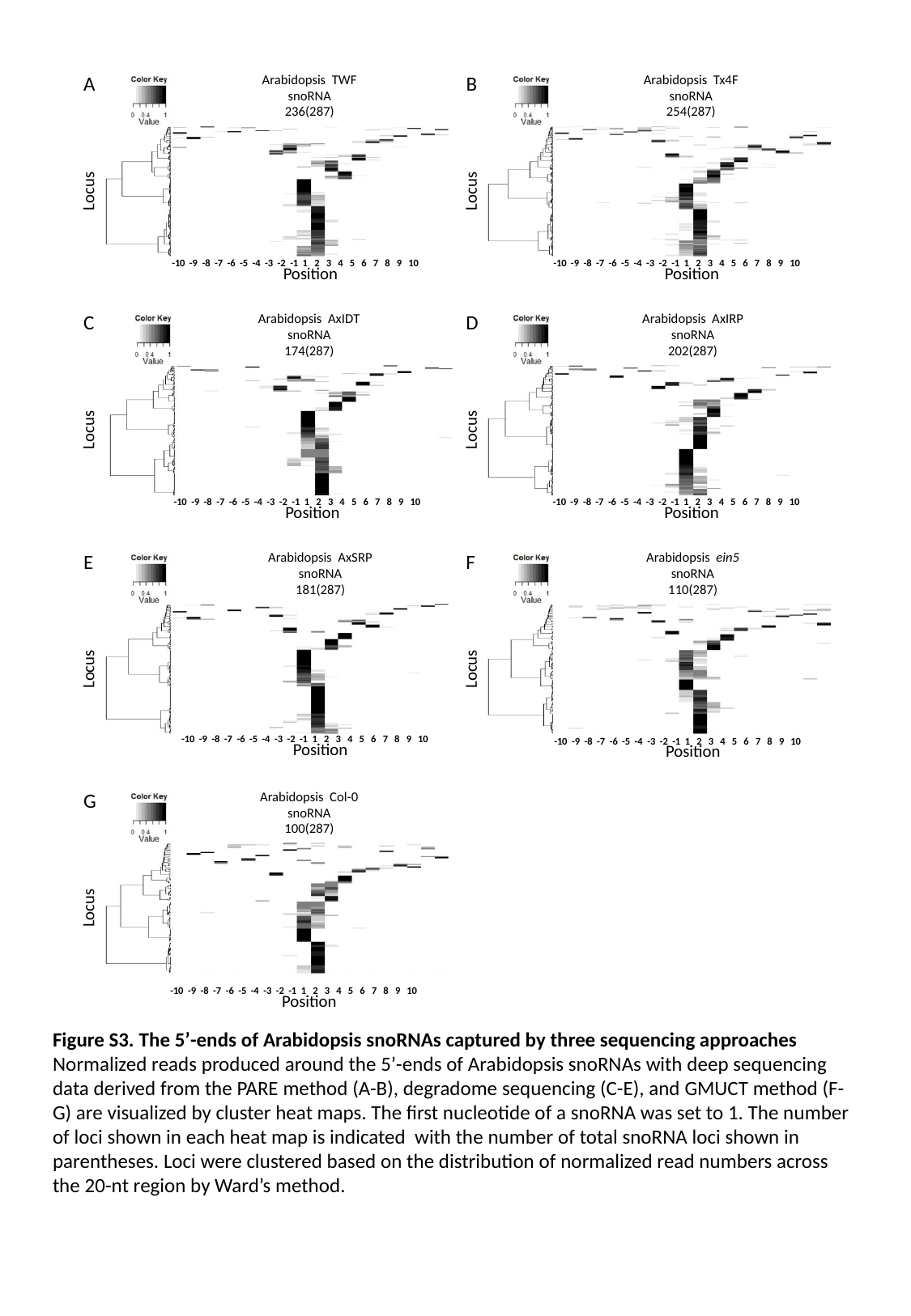

Arabidopsis TWF
snoRNA
236(287)
Locus
-10 -9 -8 -7 -6 -5 -4 -3 -2 -1 1 2 3 4 5 6 7 8 9 10
Position
A
Arabidopsis Tx4F
snoRNA
254(287)
Locus
-10 -9 -8 -7 -6 -5 -4 -3 -2 -1 1 2 3 4 5 6 7 8 9 10
Position
B
C
Arabidopsis AxIRP
snoRNA
202(287)
Locus
-10 -9 -8 -7 -6 -5 -4 -3 -2 -1 1 2 3 4 5 6 7 8 9 10
Position
D
Arabidopsis AxIDT
snoRNA
174(287)
Locus
-10 -9 -8 -7 -6 -5 -4 -3 -2 -1 1 2 3 4 5 6 7 8 9 10
Position
E
Arabidopsis AxSRP
snoRNA
181(287)
Locus
-10 -9 -8 -7 -6 -5 -4 -3 -2 -1 1 2 3 4 5 6 7 8 9 10
Position
Arabidopsis ein5
snoRNA
110(287)
Locus
-10 -9 -8 -7 -6 -5 -4 -3 -2 -1 1 2 3 4 5 6 7 8 9 10
Position
F
Arabidopsis Col-0
snoRNA
100(287)
Locus
-10 -9 -8 -7 -6 -5 -4 -3 -2 -1 1 2 3 4 5 6 7 8 9 10
Position
G
Figure S3. The 5’-ends of Arabidopsis snoRNAs captured by three sequencing approaches Normalized reads produced around the 5’-ends of Arabidopsis snoRNAs with deep sequencing data derived from the PARE method (A-B), degradome sequencing (C-E), and GMUCT method (F-G) are visualized by cluster heat maps. The first nucleotide of a snoRNA was set to 1. The number of loci shown in each heat map is indicated with the number of total snoRNA loci shown in parentheses. Loci were clustered based on the distribution of normalized read numbers across the 20-nt region by Ward’s method.

## Slide 4
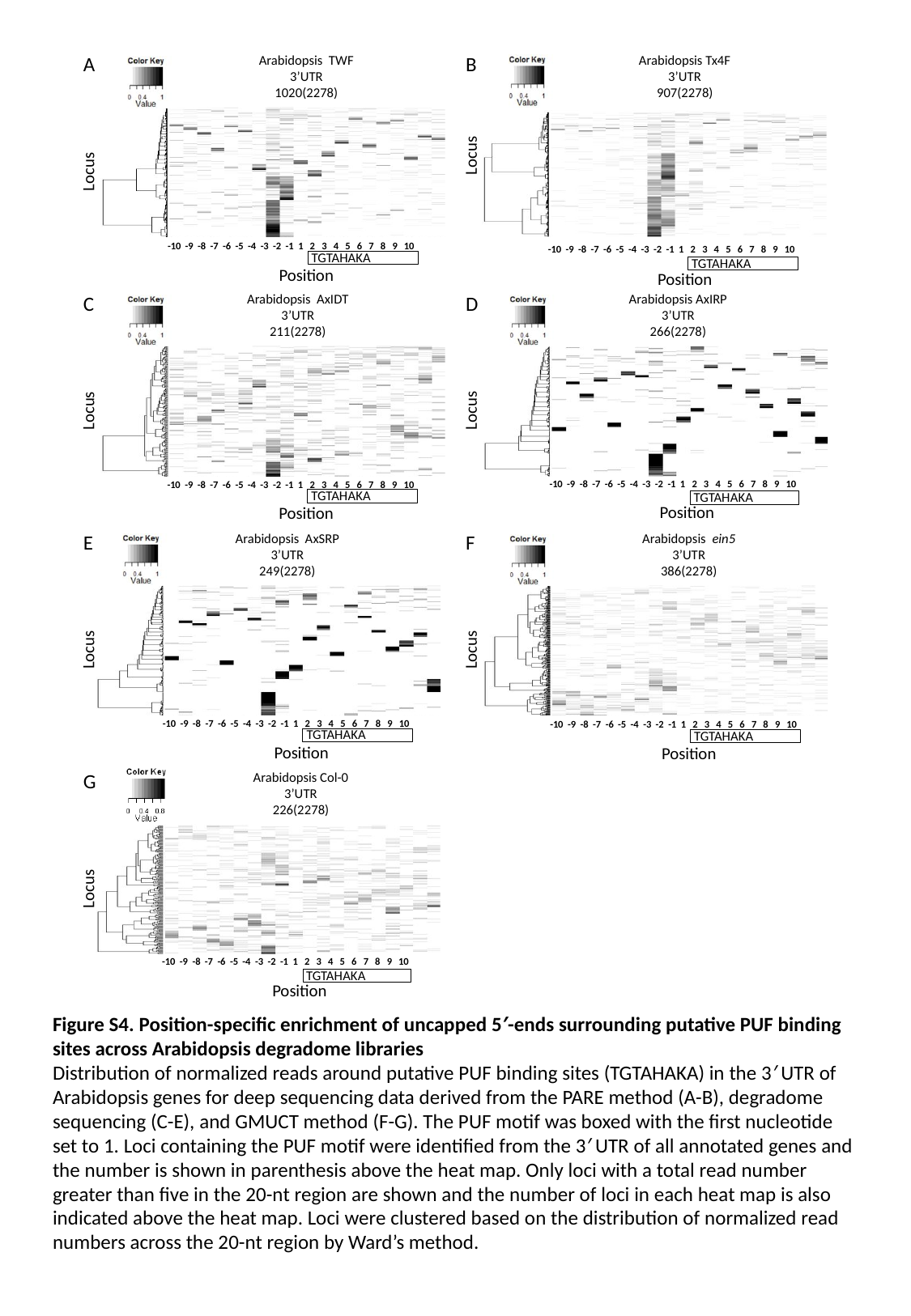

Arabidopsis TWF
3’UTR
1020(2278)
Locus
-10 -9 -8 -7 -6 -5 -4 -3 -2 -1 1 2 3 4 5 6 7 8 9 10
TGTAHAKA
Position
A
Arabidopsis Tx4F
3’UTR
907(2278)
Locus
-10 -9 -8 -7 -6 -5 -4 -3 -2 -1 1 2 3 4 5 6 7 8 9 10
TGTAHAKA
Position
B
Arabidopsis AxIDT
3’UTR
211(2278)
Locus
-10 -9 -8 -7 -6 -5 -4 -3 -2 -1 1 2 3 4 5 6 7 8 9 10
TGTAHAKA
Position
C
Arabidopsis AxIRP
3’UTR
266(2278)
Locus
-10 -9 -8 -7 -6 -5 -4 -3 -2 -1 1 2 3 4 5 6 7 8 9 10
TGTAHAKA
Position
D
Arabidopsis AxSRP
3’UTR
249(2278)
Locus
-10 -9 -8 -7 -6 -5 -4 -3 -2 -1 1 2 3 4 5 6 7 8 9 10
TGTAHAKA
Position
E
Arabidopsis ein5
3’UTR
386(2278)
Locus
-10 -9 -8 -7 -6 -5 -4 -3 -2 -1 1 2 3 4 5 6 7 8 9 10
TGTAHAKA
Position
F
Arabidopsis Col-0
3’UTR
226(2278)
Locus
-10 -9 -8 -7 -6 -5 -4 -3 -2 -1 1 2 3 4 5 6 7 8 9 10
TGTAHAKA
Position
G
Figure S4. Position-specific enrichment of uncapped 5′-ends surrounding putative PUF binding sites across Arabidopsis degradome libraries
Distribution of normalized reads around putative PUF binding sites (TGTAHAKA) in the 3′ UTR of Arabidopsis genes for deep sequencing data derived from the PARE method (A-B), degradome sequencing (C-E), and GMUCT method (F-G). The PUF motif was boxed with the first nucleotide set to 1. Loci containing the PUF motif were identified from the 3′ UTR of all annotated genes and the number is shown in parenthesis above the heat map. Only loci with a total read number greater than five in the 20-nt region are shown and the number of loci in each heat map is also indicated above the heat map. Loci were clustered based on the distribution of normalized read numbers across the 20-nt region by Ward’s method.

## Slide 5
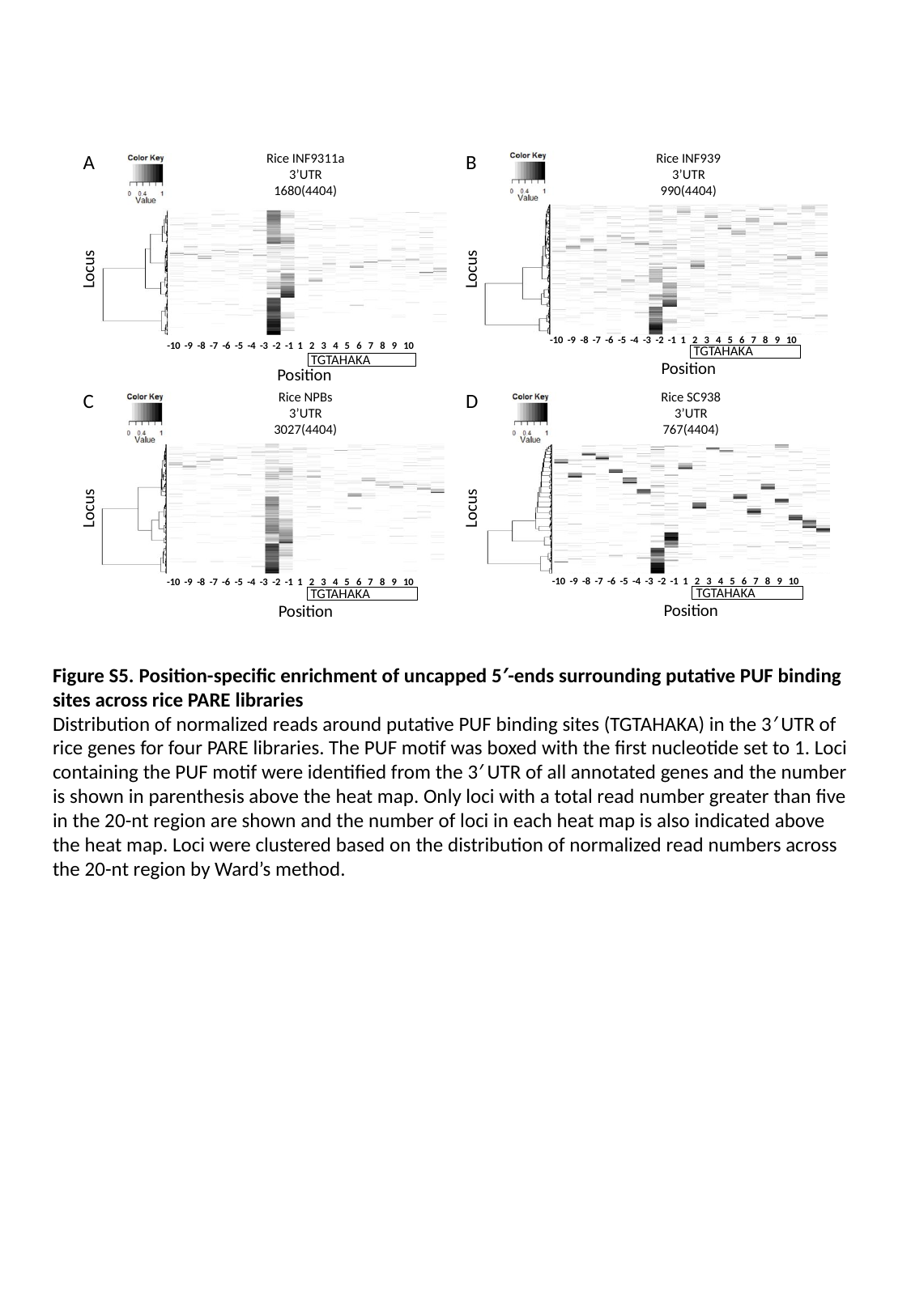

Rice INF9311a
3’UTR
1680(4404)
Locus
-10 -9 -8 -7 -6 -5 -4 -3 -2 -1 1 2 3 4 5 6 7 8 9 10
TGTAHAKA
Position
A
Rice INF939
3’UTR
990(4404)
Locus
-10 -9 -8 -7 -6 -5 -4 -3 -2 -1 1 2 3 4 5 6 7 8 9 10
TGTAHAKA
Position
B
Rice NPBs
3’UTR
3027(4404)
Locus
-10 -9 -8 -7 -6 -5 -4 -3 -2 -1 1 2 3 4 5 6 7 8 9 10
TGTAHAKA
Position
C
Rice SC938
3’UTR
767(4404)
Locus
-10 -9 -8 -7 -6 -5 -4 -3 -2 -1 1 2 3 4 5 6 7 8 9 10
TGTAHAKA
Position
D
Figure S5. Position-specific enrichment of uncapped 5′-ends surrounding putative PUF binding sites across rice PARE libraries
Distribution of normalized reads around putative PUF binding sites (TGTAHAKA) in the 3′ UTR of rice genes for four PARE libraries. The PUF motif was boxed with the first nucleotide set to 1. Loci containing the PUF motif were identified from the 3′ UTR of all annotated genes and the number is shown in parenthesis above the heat map. Only loci with a total read number greater than five in the 20-nt region are shown and the number of loci in each heat map is also indicated above the heat map. Loci were clustered based on the distribution of normalized read numbers across the 20-nt region by Ward’s method.

## Slide 6
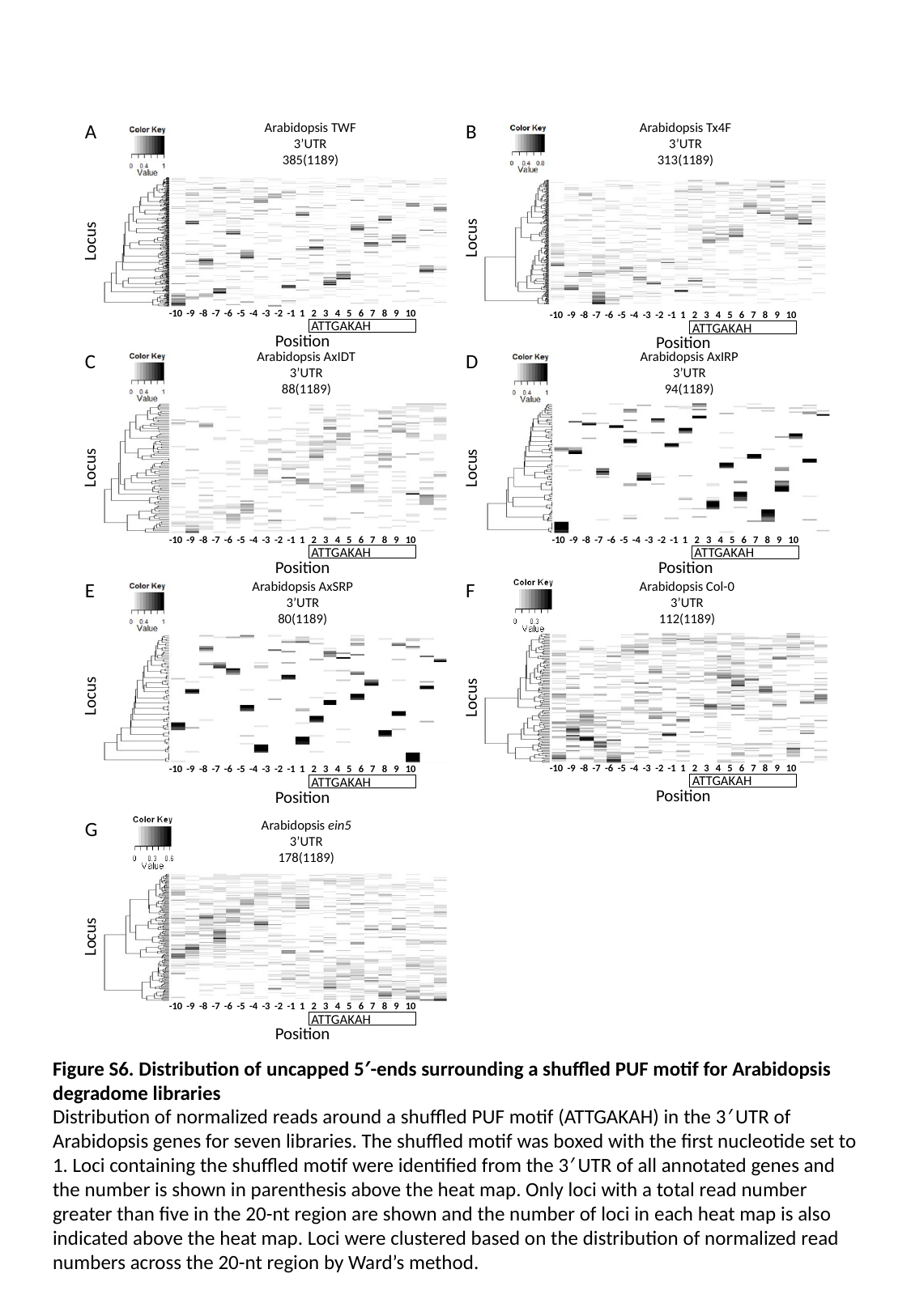

Arabidopsis TWF
3’UTR
385(1189)
Locus
-10 -9 -8 -7 -6 -5 -4 -3 -2 -1 1 2 3 4 5 6 7 8 9 10
ATTGAKAH
Position
A
Arabidopsis Tx4F
3’UTR
313(1189)
Locus
-10 -9 -8 -7 -6 -5 -4 -3 -2 -1 1 2 3 4 5 6 7 8 9 10
ATTGAKAH
Position
B
Arabidopsis AxIDT
3’UTR
88(1189)
Locus
-10 -9 -8 -7 -6 -5 -4 -3 -2 -1 1 2 3 4 5 6 7 8 9 10
ATTGAKAH
Position
C
Arabidopsis AxIRP
3’UTR
94(1189)
Locus
-10 -9 -8 -7 -6 -5 -4 -3 -2 -1 1 2 3 4 5 6 7 8 9 10
ATTGAKAH
Position
D
Arabidopsis AxSRP
3’UTR
80(1189)
Locus
-10 -9 -8 -7 -6 -5 -4 -3 -2 -1 1 2 3 4 5 6 7 8 9 10
ATTGAKAH
Position
E
Arabidopsis Col-0
3’UTR
112(1189)
Locus
-10 -9 -8 -7 -6 -5 -4 -3 -2 -1 1 2 3 4 5 6 7 8 9 10
ATTGAKAH
Position
F
Arabidopsis ein5
3’UTR
178(1189)
Locus
-10 -9 -8 -7 -6 -5 -4 -3 -2 -1 1 2 3 4 5 6 7 8 9 10
ATTGAKAH
Position
G
Figure S6. Distribution of uncapped 5′-ends surrounding a shuffled PUF motif for Arabidopsis degradome libraries
Distribution of normalized reads around a shuffled PUF motif (ATTGAKAH) in the 3′ UTR of Arabidopsis genes for seven libraries. The shuffled motif was boxed with the first nucleotide set to 1. Loci containing the shuffled motif were identified from the 3′ UTR of all annotated genes and the number is shown in parenthesis above the heat map. Only loci with a total read number greater than five in the 20-nt region are shown and the number of loci in each heat map is also indicated above the heat map. Loci were clustered based on the distribution of normalized read numbers across the 20-nt region by Ward’s method.

## Slide 7
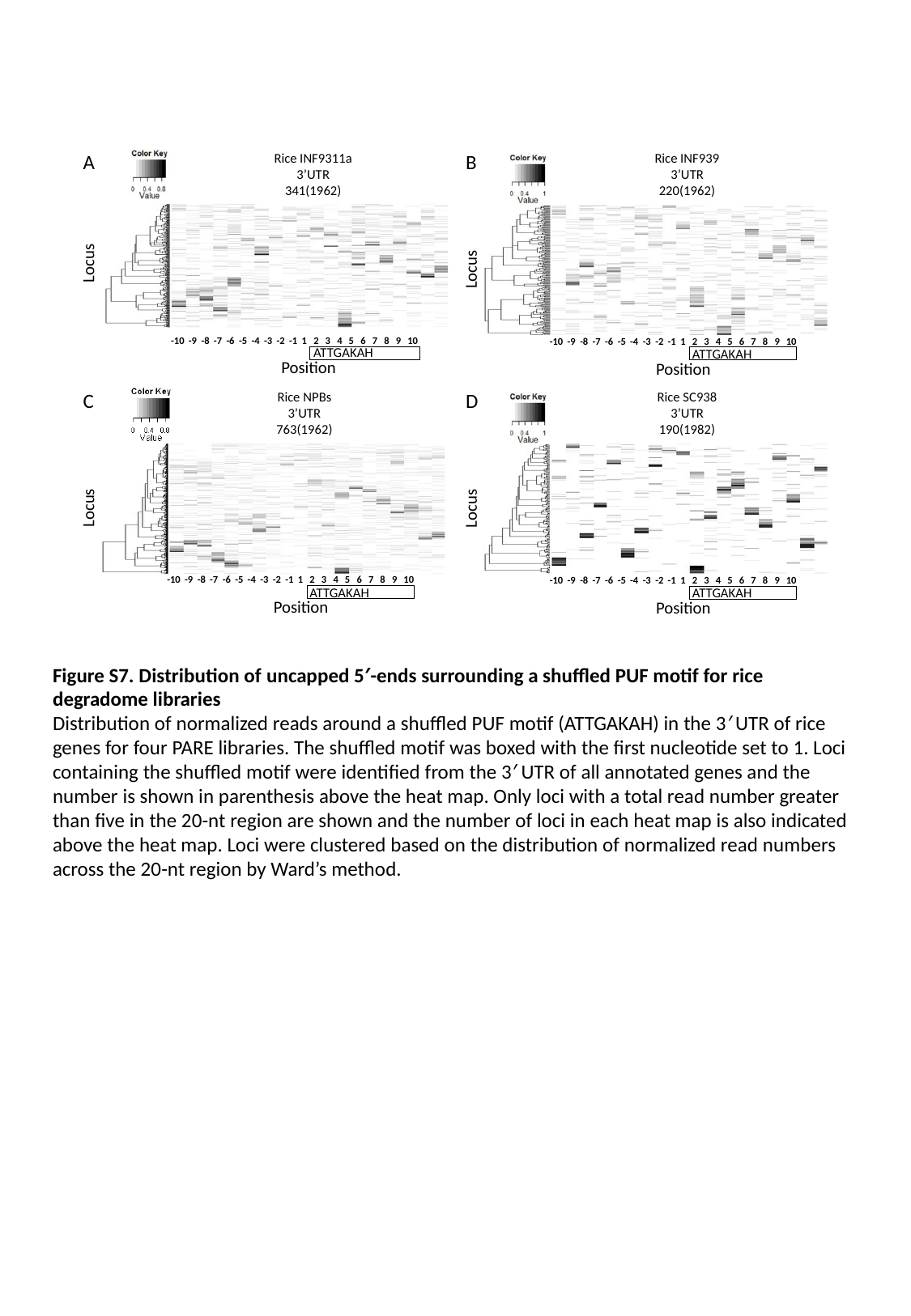

Rice INF9311a
3’UTR
341(1962)
Locus
-10 -9 -8 -7 -6 -5 -4 -3 -2 -1 1 2 3 4 5 6 7 8 9 10
ATTGAKAH
Position
A
Rice INF939
3’UTR
220(1962)
Locus
-10 -9 -8 -7 -6 -5 -4 -3 -2 -1 1 2 3 4 5 6 7 8 9 10
ATTGAKAH
Position
B
Rice NPBs
3’UTR
763(1962)
Locus
-10 -9 -8 -7 -6 -5 -4 -3 -2 -1 1 2 3 4 5 6 7 8 9 10
ATTGAKAH
Position
C
Rice SC938
3’UTR
190(1982)
Locus
-10 -9 -8 -7 -6 -5 -4 -3 -2 -1 1 2 3 4 5 6 7 8 9 10
ATTGAKAH
Position
D
Figure S7. Distribution of uncapped 5′-ends surrounding a shuffled PUF motif for rice degradome libraries
Distribution of normalized reads around a shuffled PUF motif (ATTGAKAH) in the 3′ UTR of rice genes for four PARE libraries. The shuffled motif was boxed with the first nucleotide set to 1. Loci containing the shuffled motif were identified from the 3′ UTR of all annotated genes and the number is shown in parenthesis above the heat map. Only loci with a total read number greater than five in the 20-nt region are shown and the number of loci in each heat map is also indicated above the heat map. Loci were clustered based on the distribution of normalized read numbers across the 20-nt region by Ward’s method.

## Slide 8
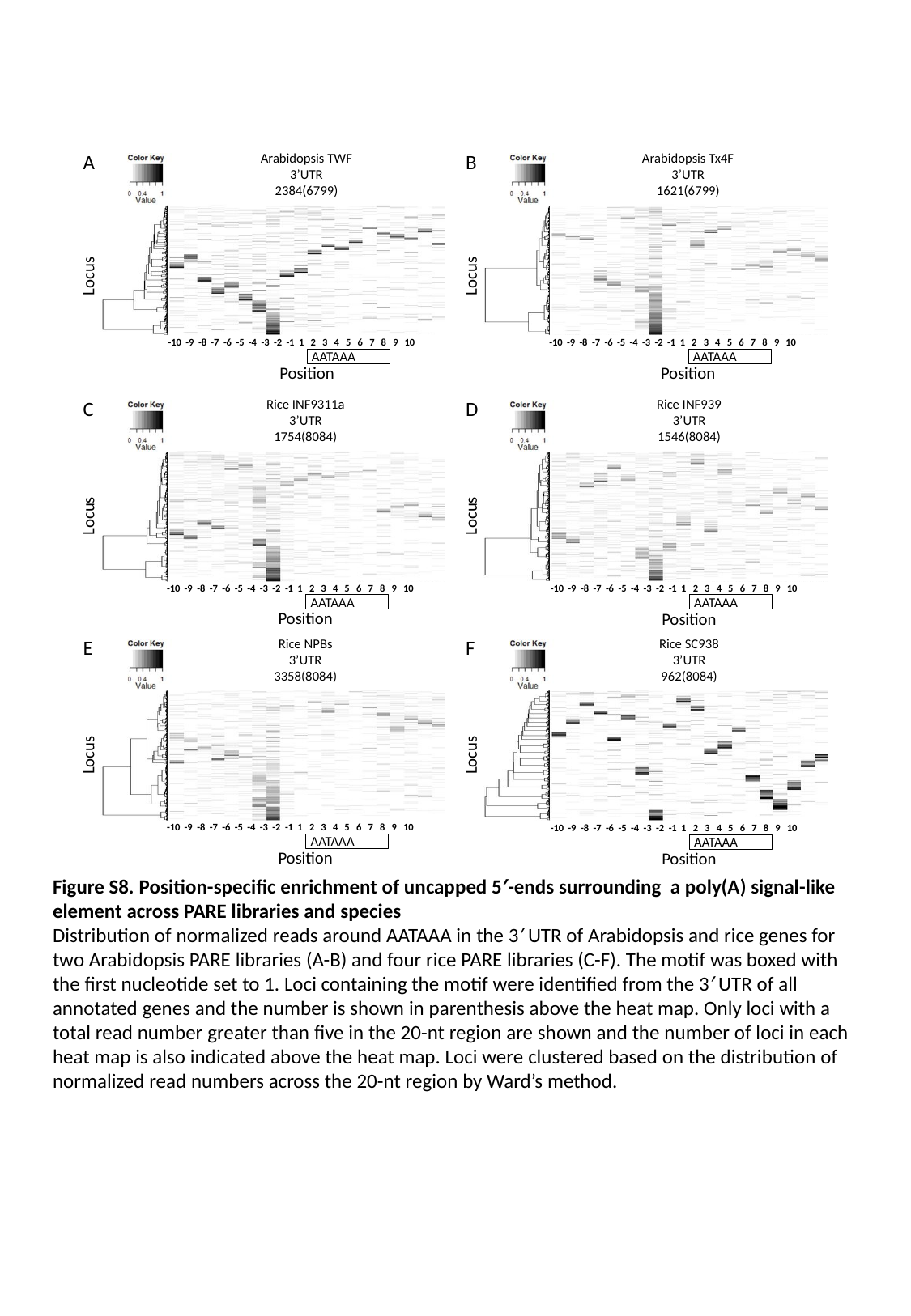

Arabidopsis TWF
3’UTR
2384(6799)
Locus
-10 -9 -8 -7 -6 -5 -4 -3 -2 -1 1 2 3 4 5 6 7 8 9 10
AATAAA
Position
A
Arabidopsis Tx4F
3’UTR
1621(6799)
Locus
-10 -9 -8 -7 -6 -5 -4 -3 -2 -1 1 2 3 4 5 6 7 8 9 10
AATAAA
Position
B
Rice INF9311a
3’UTR
1754(8084)
Locus
-10 -9 -8 -7 -6 -5 -4 -3 -2 -1 1 2 3 4 5 6 7 8 9 10
AATAAA
Position
C
Rice INF939
3’UTR
1546(8084)
Locus
-10 -9 -8 -7 -6 -5 -4 -3 -2 -1 1 2 3 4 5 6 7 8 9 10
AATAAA
Position
D
Rice NPBs
3’UTR
3358(8084)
Locus
-10 -9 -8 -7 -6 -5 -4 -3 -2 -1 1 2 3 4 5 6 7 8 9 10
AATAAA
Position
E
Rice SC938
3’UTR
962(8084)
Locus
-10 -9 -8 -7 -6 -5 -4 -3 -2 -1 1 2 3 4 5 6 7 8 9 10
AATAAA
Position
F
Figure S8. Position-specific enrichment of uncapped 5′-ends surrounding a poly(A) signal-like element across PARE libraries and species
Distribution of normalized reads around AATAAA in the 3′ UTR of Arabidopsis and rice genes for two Arabidopsis PARE libraries (A-B) and four rice PARE libraries (C-F). The motif was boxed with the first nucleotide set to 1. Loci containing the motif were identified from the 3′ UTR of all annotated genes and the number is shown in parenthesis above the heat map. Only loci with a total read number greater than five in the 20-nt region are shown and the number of loci in each heat map is also indicated above the heat map. Loci were clustered based on the distribution of normalized read numbers across the 20-nt region by Ward’s method.

## Slide 9
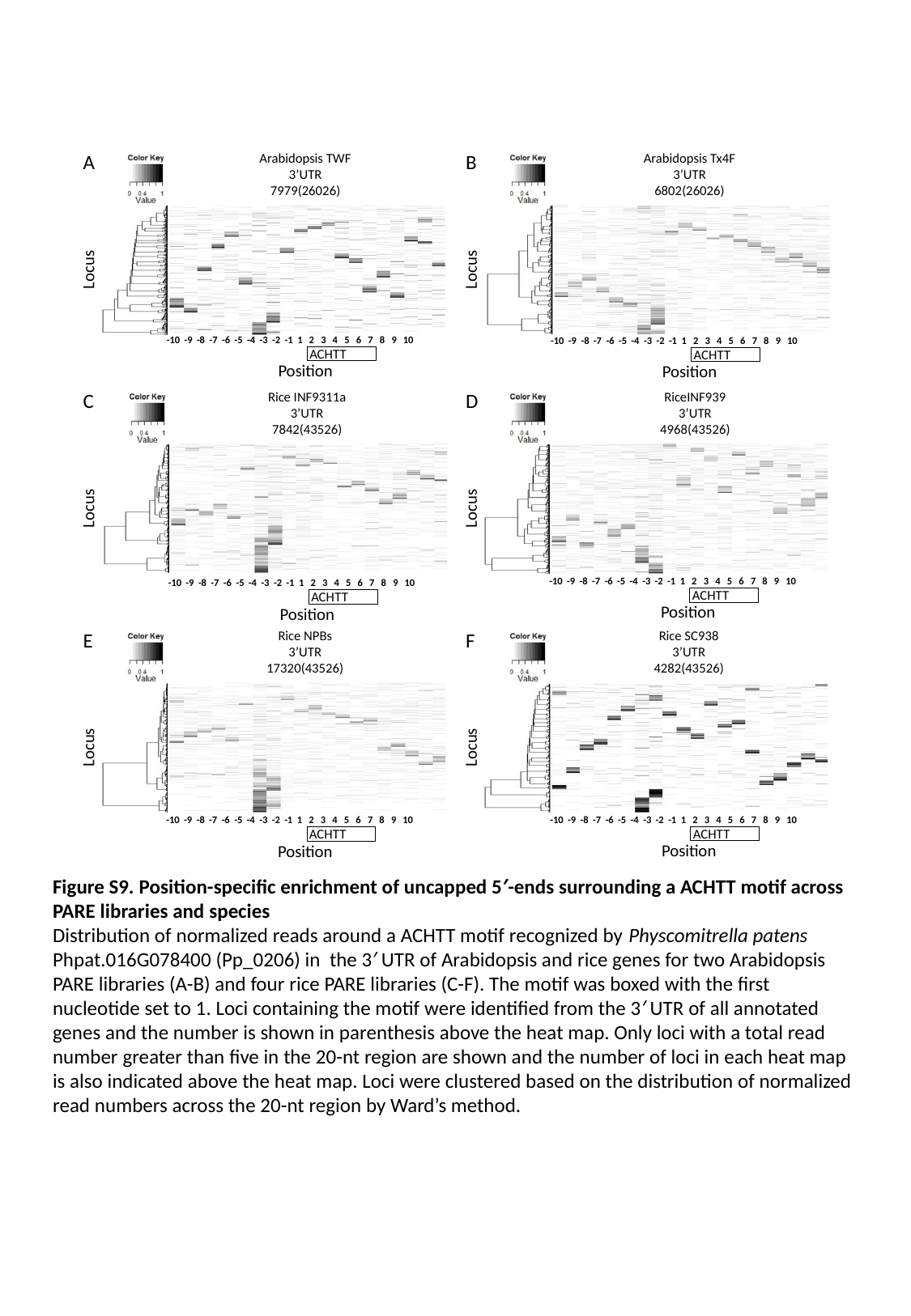

Arabidopsis TWF
3’UTR
7979(26026)
Locus
-10 -9 -8 -7 -6 -5 -4 -3 -2 -1 1 2 3 4 5 6 7 8 9 10
ACHTT
Position
A
Arabidopsis Tx4F
3’UTR
6802(26026)
Locus
-10 -9 -8 -7 -6 -5 -4 -3 -2 -1 1 2 3 4 5 6 7 8 9 10
ACHTT
Position
B
Rice INF9311a
3’UTR
7842(43526)
Locus
-10 -9 -8 -7 -6 -5 -4 -3 -2 -1 1 2 3 4 5 6 7 8 9 10
ACHTT
Position
C
RiceINF939
3’UTR
4968(43526)
Locus
-10 -9 -8 -7 -6 -5 -4 -3 -2 -1 1 2 3 4 5 6 7 8 9 10
ACHTT
Position
D
Rice SC938
3’UTR
4282(43526)
Locus
-10 -9 -8 -7 -6 -5 -4 -3 -2 -1 1 2 3 4 5 6 7 8 9 10
ACHTT
Position
Rice NPBs
3’UTR
17320(43526)
Locus
-10 -9 -8 -7 -6 -5 -4 -3 -2 -1 1 2 3 4 5 6 7 8 9 10
ACHTT
Position
E
F
Figure S9. Position-specific enrichment of uncapped 5′-ends surrounding a ACHTT motif across PARE libraries and species
Distribution of normalized reads around a ACHTT motif recognized by Physcomitrella patens Phpat.016G078400 (Pp_0206) in the 3′ UTR of Arabidopsis and rice genes for two Arabidopsis PARE libraries (A-B) and four rice PARE libraries (C-F). The motif was boxed with the first nucleotide set to 1. Loci containing the motif were identified from the 3′ UTR of all annotated genes and the number is shown in parenthesis above the heat map. Only loci with a total read number greater than five in the 20-nt region are shown and the number of loci in each heat map is also indicated above the heat map. Loci were clustered based on the distribution of normalized read numbers across the 20-nt region by Ward’s method.

## Slide 10
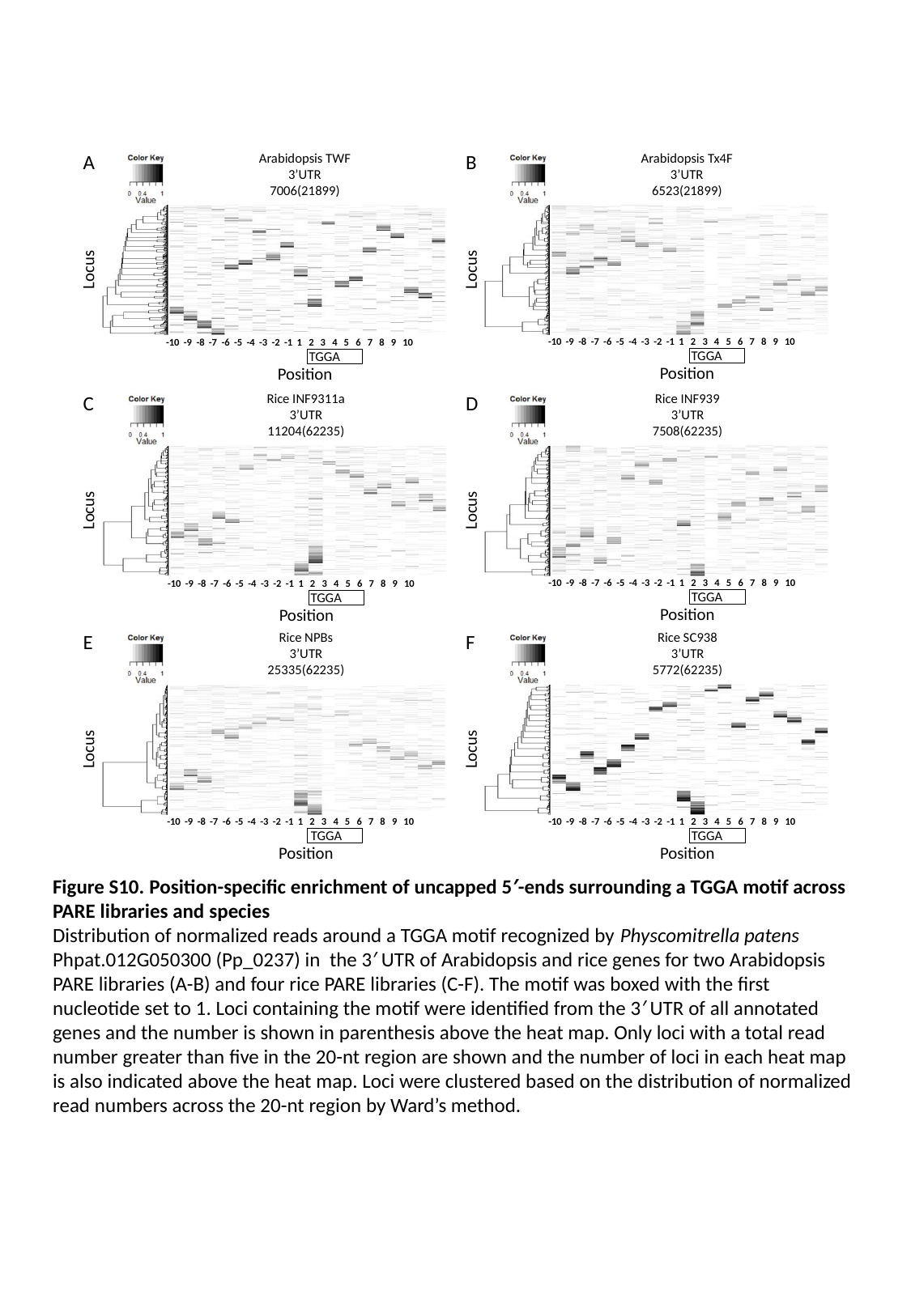

Arabidopsis Tx4F
3’UTR
6523(21899)
Locus
-10 -9 -8 -7 -6 -5 -4 -3 -2 -1 1 2 3 4 5 6 7 8 9 10
TGGA
Position
A
Arabidopsis TWF
3’UTR
7006(21899)
Locus
-10 -9 -8 -7 -6 -5 -4 -3 -2 -1 1 2 3 4 5 6 7 8 9 10
TGGA
Position
B
Rice INF9311a
3’UTR
11204(62235)
Locus
-10 -9 -8 -7 -6 -5 -4 -3 -2 -1 1 2 3 4 5 6 7 8 9 10
TGGA
Position
C
Rice INF939
3’UTR
7508(62235)
Locus
-10 -9 -8 -7 -6 -5 -4 -3 -2 -1 1 2 3 4 5 6 7 8 9 10
TGGA
Position
D
Rice NPBs
3’UTR
25335(62235)
Locus
-10 -9 -8 -7 -6 -5 -4 -3 -2 -1 1 2 3 4 5 6 7 8 9 10
TGGA
Position
E
Rice SC938
3’UTR
5772(62235)
Locus
-10 -9 -8 -7 -6 -5 -4 -3 -2 -1 1 2 3 4 5 6 7 8 9 10
TGGA
Position
F
Figure S10. Position-specific enrichment of uncapped 5′-ends surrounding a TGGA motif across PARE libraries and species
Distribution of normalized reads around a TGGA motif recognized by Physcomitrella patens Phpat.012G050300 (Pp_0237) in the 3′ UTR of Arabidopsis and rice genes for two Arabidopsis PARE libraries (A-B) and four rice PARE libraries (C-F). The motif was boxed with the first nucleotide set to 1. Loci containing the motif were identified from the 3′ UTR of all annotated genes and the number is shown in parenthesis above the heat map. Only loci with a total read number greater than five in the 20-nt region are shown and the number of loci in each heat map is also indicated above the heat map. Loci were clustered based on the distribution of normalized read numbers across the 20-nt region by Ward’s method.

## Slide 11
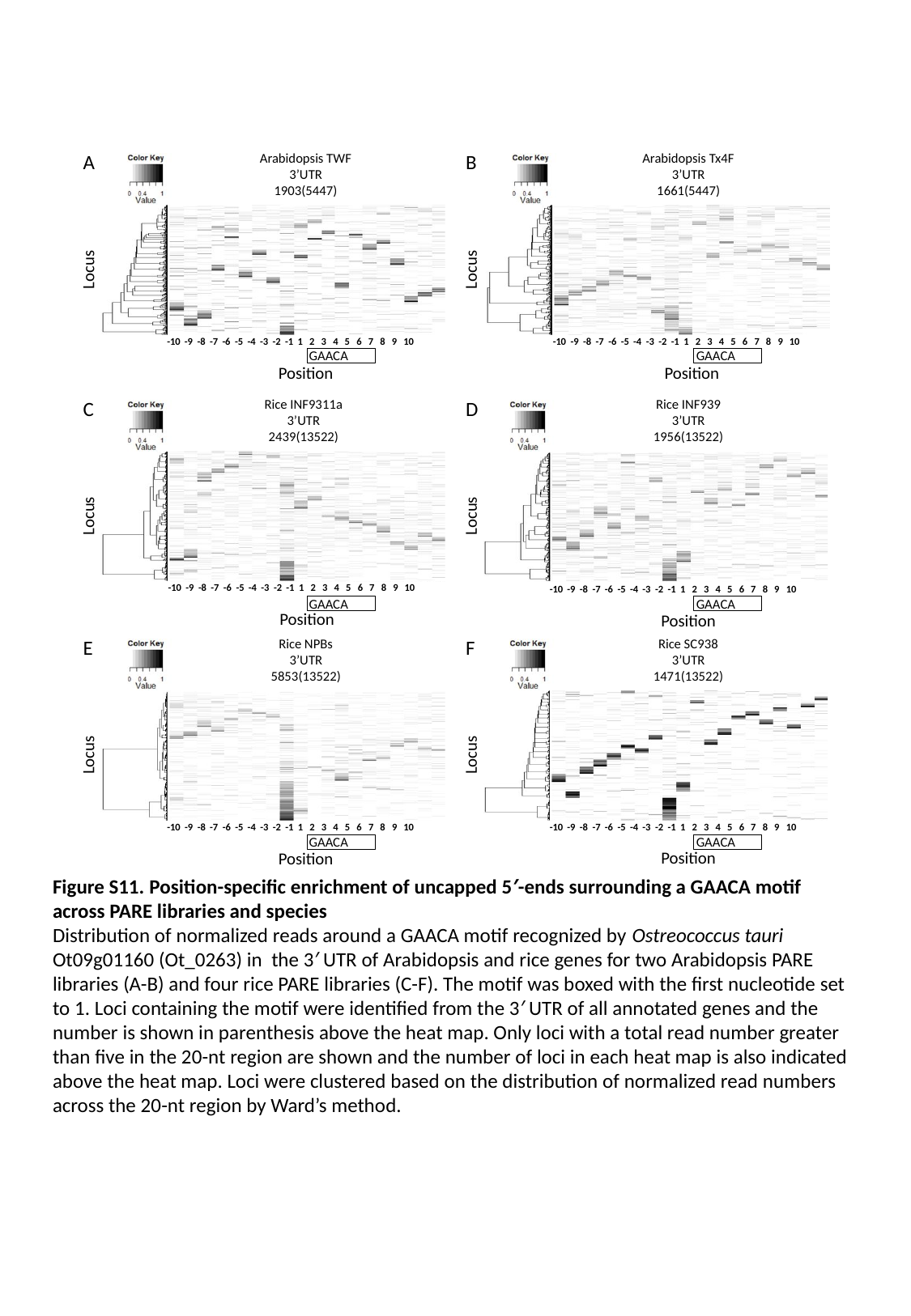

Arabidopsis TWF
3’UTR
1903(5447)
Locus
-10 -9 -8 -7 -6 -5 -4 -3 -2 -1 1 2 3 4 5 6 7 8 9 10
GAACA
Position
A
B
Arabidopsis Tx4F
3’UTR
1661(5447)
Locus
-10 -9 -8 -7 -6 -5 -4 -3 -2 -1 1 2 3 4 5 6 7 8 9 10
GAACA
Position
C
Rice INF9311a
3’UTR
2439(13522)
Locus
-10 -9 -8 -7 -6 -5 -4 -3 -2 -1 1 2 3 4 5 6 7 8 9 10
GAACA
Position
D
Rice INF939
3’UTR
1956(13522)
Locus
-10 -9 -8 -7 -6 -5 -4 -3 -2 -1 1 2 3 4 5 6 7 8 9 10
GAACA
Position
E
Rice NPBs
3’UTR
5853(13522)
Locus
-10 -9 -8 -7 -6 -5 -4 -3 -2 -1 1 2 3 4 5 6 7 8 9 10
GAACA
Position
F
Rice SC938
3’UTR
1471(13522)
Locus
-10 -9 -8 -7 -6 -5 -4 -3 -2 -1 1 2 3 4 5 6 7 8 9 10
GAACA
Position
Figure S11. Position-specific enrichment of uncapped 5′-ends surrounding a GAACA motif across PARE libraries and species
Distribution of normalized reads around a GAACA motif recognized by Ostreococcus tauri Ot09g01160 (Ot_0263) in the 3′ UTR of Arabidopsis and rice genes for two Arabidopsis PARE libraries (A-B) and four rice PARE libraries (C-F). The motif was boxed with the first nucleotide set to 1. Loci containing the motif were identified from the 3′ UTR of all annotated genes and the number is shown in parenthesis above the heat map. Only loci with a total read number greater than five in the 20-nt region are shown and the number of loci in each heat map is also indicated above the heat map. Loci were clustered based on the distribution of normalized read numbers across the 20-nt region by Ward’s method.

## Slide 12
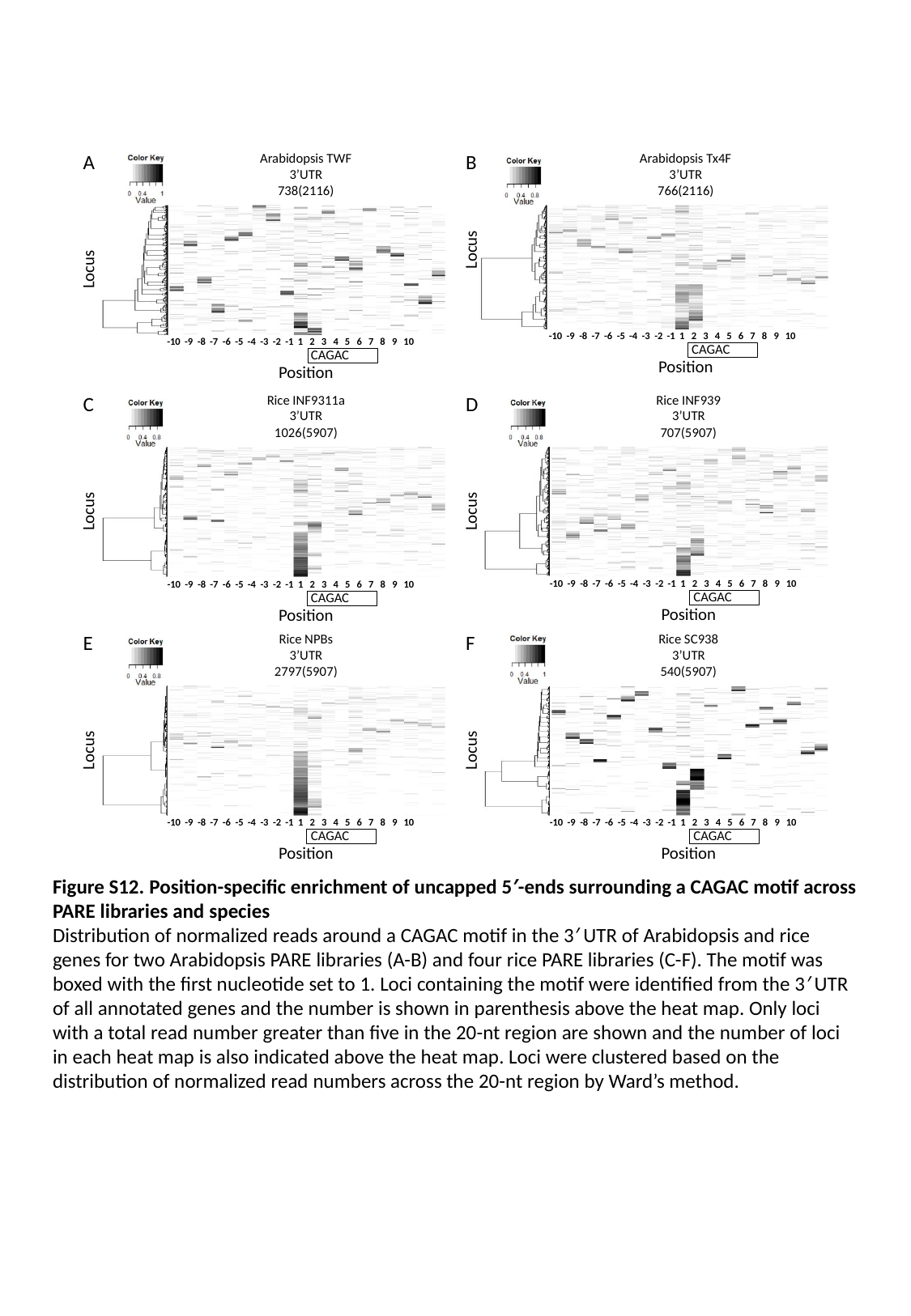

Arabidopsis TWF
3’UTR
738(2116)
Locus
-10 -9 -8 -7 -6 -5 -4 -3 -2 -1 1 2 3 4 5 6 7 8 9 10
CAGAC
Position
A
Arabidopsis Tx4F
3’UTR
766(2116)
Locus
-10 -9 -8 -7 -6 -5 -4 -3 -2 -1 1 2 3 4 5 6 7 8 9 10
CAGAC
Position
B
Rice INF9311a
3’UTR
1026(5907)
Locus
-10 -9 -8 -7 -6 -5 -4 -3 -2 -1 1 2 3 4 5 6 7 8 9 10
CAGAC
Position
C
Rice INF939
3’UTR
707(5907)
Locus
-10 -9 -8 -7 -6 -5 -4 -3 -2 -1 1 2 3 4 5 6 7 8 9 10
CAGAC
Position
D
Rice NPBs
3’UTR
2797(5907)
Locus
-10 -9 -8 -7 -6 -5 -4 -3 -2 -1 1 2 3 4 5 6 7 8 9 10
CAGAC
Position
E
Rice SC938
3’UTR
540(5907)
Locus
-10 -9 -8 -7 -6 -5 -4 -3 -2 -1 1 2 3 4 5 6 7 8 9 10
CAGAC
Position
F
Figure S12. Position-specific enrichment of uncapped 5′-ends surrounding a CAGAC motif across PARE libraries and species
Distribution of normalized reads around a CAGAC motif in the 3′ UTR of Arabidopsis and rice genes for two Arabidopsis PARE libraries (A-B) and four rice PARE libraries (C-F). The motif was boxed with the first nucleotide set to 1. Loci containing the motif were identified from the 3′ UTR of all annotated genes and the number is shown in parenthesis above the heat map. Only loci with a total read number greater than five in the 20-nt region are shown and the number of loci in each heat map is also indicated above the heat map. Loci were clustered based on the distribution of normalized read numbers across the 20-nt region by Ward’s method.
